# *Chrysanthemum*: A Comprehensive Review on Recent Developments on In Vitro Regeneration

**DOI:** 10.3390/biology11121774

**Published:** 2022-12-06

**Authors:** Eman Abdelhakim Eisa, Andrea Tilly-Mándy, Péter Honfi, Awad Yousef Shala, Mayank Anand Gururani

**Affiliations:** 1 Department of Floriculture and Dendrology, Hungarian University of Agriculture and Life Science (MATE), 1118 Budapest, Hungary; 2 Botanical Gardens Research Department, Horticulture Research Institute, Agricultural Research Center (ARC), Giza 12619, Egypt; 3 Medicinal and Aromatic Plants Research Department, Horticulture Research Institute, Agricultural Research Center (ARC), Giza 12619, Egypt; 4 Biology Department, College of Science, United Arab Emirates University, Al Ain P.O. Box 15551, United Arab Emirates

**Keywords:** *Chrysanthemum*, in vitro propagation, true-to-type plants, explant sources, medium compositions, alternative disinfections, mutagenesis, acclimatization, alternative light sources

## Abstract

**Simple Summary:**

Chrysanthemums are the second most important floricultural (cut-flower) crop after roses. A very well-studied topic, in vitro propagation of Chrysanthemums is making significant strides every year. An overview of in vitro propagation of *Chrysanthemum* in the wider plant science literature is presented in this review. The review presents a comprehensive understanding of the recently described methods of sterilization, plant growth regulators, hormonal combinations, acclimation efficiency, and light conditions for the rapid and cost-effective in vitro regeneration of *Chrysanthemum* in the last decade.

**Abstract:**

*Chrysanthemum* is a flowering plant grown worldwide and is one of the most popular ornamental plants. Chrysanthemums are usually cultivated using root suckers and shoot cuttings. This conventional technique is relatively slow. In addition, as cuttings are gained regularly from mother plants, there is a chance of viral infection and degeneration, which raises the production cost. The hurdles mentioned above have been managed by applying in vitro propagation techniques, which can enhance reproduction rates through in vitro culture and use very small explants, which are impossible with the conventional approach. Usually, it is difficult to get true-to-type plants as the parents with good quality, but clonal propagation of a designated elite species makes it possible. Hence, this review highlights recent studies of the in vitro propagation of *Chrysanthemum* included; the appropriate explant sources, medium compositions, alternative disinfection of culture media, plant growth regulators (PGRs), different mutagenesis applications, acclimatization efficiency, and alternative light sources to overcome the shortcomings of conventional propagation techniques.

## 1. Introduction

The floriculture industry is considered one of the most important and rapidly growing commercial trades in the agriculture industry. Many cut flowers and potted plants are being sold worldwide, for instance (*Alstroemeria*, *Anthurium*, carnation, *Chrysanthemum*, *Gerbera*, *Gladiolus*, *Lilium*, Lisianthus, and roses) on a daily basis [1]. The world’s top countries with value-wise production and export of cut plants are the Netherlands, the United States, Japan, Italy, Germany, and Canada. Germany, the United States, France, and the United Kingdom are the leading consumer countries [2].

**Figure 1:**
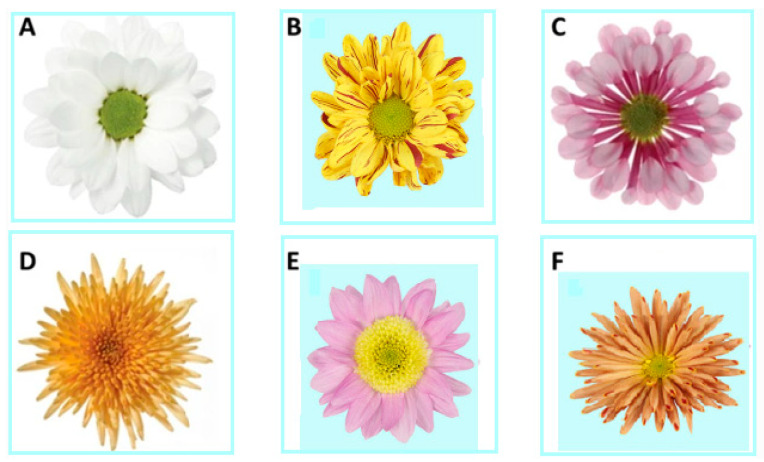
(**A**) The wild-type *Chrysanthemum* flower shape; (**B**) the double flower type, in which all of the disc florets have turned into the ray florets; (**C**) a spoon type is produced by partially united ray floret petals; (**D**) in combination with the double type, fully united ray florets produce a spider type; (**E**) a form of anemone with colorful and large disc floret petals.; (**F**) An arrangement of totally joined ray floret petals and sizable disc floret petals is known as a spider-anemone. Modified after, Spaargaren and Geest, [4].

The term ‘chrysanthemum’ comes from the Greek ‘krus anthemon’, which means gold flower, and was initially used in China. *Chrysanthemum morifolium (Ramat),* belonging to the Asteraceae family [3], is considered the second most important floricultural crop worldwide after roses [4]. This is a culturally significant flower with an annual sale of billions of branches. It is propagated in diverse colors, sizes, and forms of composite *Chrysanthemum* flowers by collecting several combinations, concentrations, and types of anthocyanins (purple), carotenoids (yellow), and chlorophyll (green) (Figure 1).

*Chrysanthemum* is one of the most utilized plants in traditional medicine. The flower reduces inflammation and treats bruises, sprains, bites from snakes and centipedes, rhinitis, diphtheria, cholera, and malaria [5]. It also has antipyretic and antihypertensive features [6]. *Chrysanthemum* petals have been used to treat various diseases, such as fever and wind-heat syndrome [7]. Chinese people eat flowers as a salad, and dried petals are used to make tea (tisane) [8].

Most *Chrysanthemum* species belong to East Asia. China and Japan have the largest covered areas for production, with 8475 ha (2013) and 5230 ha (2009), respectively. Thailand and India are particularly prominent for domestic market sellers, with 19,000 ha and 2199 ha, respectively. A 2365 ha open production area in Mexico was mentioned in 2012 [9]. There is a constant demand and need for new varieties in the modern horticulture industry, specifically from the cut flower industry. Introducing innovative qualities in plant appearances, such as flower color/inflorescences, flower shape, plant architecture, and foliage variation, is the main goal of ornamental breeding [10].

Plant tissue culture is an in vitro aseptic cultivation of plant cells, tissues, organs, embryos, protoplasts, or seeds on a nutritional medium in a controlled environment in which humidity, temperature, light, photoperiod, and the nutrient medium are the contributing factors for an optimum growing environment. Micropropagation of healthy plants gives faster reproduction rates in a short time [11].

Popularity and demand have made *Chrysanthemum* one of the first commercial targets for micropropagation, allowing the use of tissue culture in the mass production of this flower. The primary method of *Chrysanthemum* propagation is generally done vegetatively with shoot cuttings and root suckers. This conventional process is simple, economical, and can be done in vitro. However, there are limitations to this strategy, such as a low reproduction rate, poor quality of seedlings, higher reproducible time, seasonal constraints, inadequate gene pool, and the inability to avoid cross-incompatibility; additionally, cuttings obtained frequently from mother plants may become infected with viruses and degenerate, which would increase production expenses [12]. These limitations may be modified easily with the available methods of induced mutagenesis and regeneration, in addition to an appropriate, mutable mother plant [13,14]. Hence, there is a requirement for a more effective propagation system. Micropropagation is a rapid and productive way to generate plants on a larger scale to obtain flowers for pyrethrin extraction. In a study by Catalano et al. [15], which presented the results of a 9-year field comparison between pyrethrum plants from conventional and micro-propagated methods, which were characterized by similar field performance, in both cases allowing long-lasting stands with satisfactory yields. However, reduced technical inputs were applied [4]. However, multiple factors, including medium composition, the interaction between growth regulators, explant type, plant genotype, and explant stage of development, influence the success of *Chrysanthemum* in vitro propagation [16]. Various literature studies have mentioned using tissue culture to experiment with the large-scale propagation of *C. morifolium* by utilizing various novel regeneration pathways [14,17,18,19,20,21,22,23,24]. Furthermore, establishing strategies to inhibit microbial contamination in culture media is the optimum procedure. Physical sterilization by autoclave can be substituted by applying chemicals, nanoparticles, or plant extracts alone or by combining autoclaving [25]. Moreover, explants in vitro are an extraordinarily suitable irradiation material. Thanks to the application of in vitro cultures on a small area, in a disease- and pest-free environment, a large number of irradiated explants could be placed, which ensures a more effective regeneration than in vivo conditions, increases the probability of obtaining mutated plants, and permits a significant acceleration of all stages of the breeding program [26]. In earlier studies [26,27], a physical mutagen (gamma rays) was used to induce mutations in *Chrysanthemum* in vitro and modifications in inflorescence and cultivar output were noticed. The accomplishment of successful acclimation in nursery circumstances is of critical importance to the accomplishment of successful micropropagation procedures. Plants’ survival rates are significantly lower in the acclimatization stage than in the in vitro growth stage due to exposure to several environmental stressors, such as light quality and microbial contamination, which poses a substantial challenge for micropropagation, resulting in poor plant quality and the loss of valuable plant stocks. On the other hand, artificial light in the greenhouse and in vitro could be used to regulate plant development. Compared to conventional fluorescent lamps, light-emitting diodes (LEDs), LED-Uni-Pack (LP), and wireless power transmission (WPT-LP) have emerged as alternate light sources for the optimal growth and development of *Chrysanthemum* in vitro and ex vitro [28,29]. Therefore, the current review aims to observe the recent methods in the last decade on sterilization methods, plant growth regulators (PGRs), and the best hormonal combination, acclimation efficiency, and light conditions to establish a technique for the in vitro regeneration of *Chrysanthemum* as a rapid and cost-effective approach.

## 2. In Vitro Plantlet Propagation

Several parts of plants, such as seeds, cuttings, tubers, roots, anthers, pollen, and even leaves, can be used to propagate *Chrysanthemum* in vitro. Leaves could be used as starting materials and seeds to propagate the plant. But vegetative propagation (cuttings, suckers) is favored due to its high heterozygosity and for commercial purposes. However, reproduction is too slow to be commercially viable by this approach [30]. 

In the micropropagation strategy, “cloning” refers to replicating huge numbers of selected plants with the same genotype as their parent plant through culture [31]. Anderson, in 1980, ref. [32], performed a study wherein he described the five steps of micropropagation: selecting a stock plant, establishing, reproducing, pretransplant/rooting, and finally, transplantation.

Considering the fact that successful procedures developed for one cultivar are not simply adapted to another cultivar, breeders of chrysanthemums face a difficult challenge each year: coming up with several novel and marketable cultivars as rapidly as possible. To effectively improve *Chrysanthemum* cultivars for crop production, it is necessary to create regeneration methods. Thrope [33] compiled a list of cultivar traits thought critical for the success of the morphogenesis process in vitro regeneration. The following factors were considered: (a) the source organ chosen to be utilized for tissue culture, (b) the physiological and ontogenetic age of the chosen organ, (c) the optimum season for acquiring explants, (d) explant size, and (e) the overall quality of the plant from which explants were obtained. From past studies [17,18,34], it was found that many factors that influenced *Chrysanthemum* shoot regeneration in vitro consisted of interacting with the genetic structure of the plant, type of explant, gelling agents, ethylene inhibitors, darkness period, and regulatory factors for plant growth. 

The 15 years of research on reproduction in stem segment culture [35] showed that field experiments on clonal fidelity are cultivar-specific for *Chrysanthemum* cultivars. The clonal fidelity of flowers was higher in spider-type Chrysanthemums than in daisy-type cultivars. These results have economic implications since they allow for continuous micropropagation via the long-term tissue culture multiplication of the investigated cultivars of the spider type without evident changes to flower morphology. On the contrary, our findings suggested that *Chrysanthemum* cultivars may be propagated long-term to develop economically viable cultivars. 

Waseem et al. [36] have researched different explant types impacting callus induction and organogenesis. The explant types include leaf bits, stem discs [37], leaf [38,39], pedicle [40], protoplast [41], shoot buds, top buds and axillary buds [42], stems [43], and stems with axillary buds [44]. Further, Khan [45] also stated that leaf, petiole, and stem *Chrysanthemum* cv. *nankingense* could be utilized to design plant regeneration technology. 

### 2.1. Propagation from Axillary Buds 

The ability to regenerate a large number of shoots from cultured tissues is important for the success of most in vitro propagation techniques. The capacity of *Chrysanthemum’s* shoot apex and nodal explants to regenerate in vitro is overall documented. The culture of nodal segments containing axillary buds involves the exploitation of buds already existing on the parent stock plant, hence providing an efficient method of rapid clonal proliferation enabling the creation of genetically stable and true-to-type progeny [46]. According to [47], the nodal segments of *Chrysanthemum morifulium* L. have been used to design an effective plant regeneration method [48]. Single nodal cuttings can be considered possible propagules for the generation of *Chrysanthemum* plants on a larger scale from tissue culture. A similar study on single nodal cuttings was done wherein it was with an intact leaf and dipped in Hormex solution for 10–15 min, showing excellent survival (93 to 100%) at four weeks, irrespective of treatment, and 100% root growth was also seen, but the number of roots increased in control and decreased with longer dipping times. On the contrary, cuttings without an intact leaf did not respond well to treatments because of low survivability (50%), and only 7–13% of roots could survive. An earlier study by Zalewska et al. [49] performed an experiment wherein five cultivars of *Chrysanthemum* were cultivated on MS media, having three shoot zones: distal, middle, and proximal. Two single-node explants were extracted from each zone and grown on MS media without any growth regulator supplements. After 10 weeks of cultivation, axillary buds were found to have 50% shoot growth, while the remaining shoots could be grown in rooting media with 0.2 mg/L of IBA. 

### 2.2. Propagation from Adventitious Shoots or Embryos

#### Direct and Indirect Morphogenesis

Direct morphogenesis: New cultivars with unique traits can be regenerated from disc or ray florets by forming adventitious shoots or somatic embryogenesis, a chimeric (or mutant) form in regenerated plants [50,51]. There is a vast potential for commercial *Chrysanthemum* floriculture on an industrial scale in direct floret regeneration methodology [52]. A direct regeneration strategy is preferred to preserve genetic fidelity, as demonstrated by *Chrysanthemum* cv.’s disc and ray florets. For commercial use, ‘Kargil 99′ and other mini variations have been standardized for large-scale direct organogenesis multiplication [30]. Cultures of regenerating ligulate florets increased the number of regenerating shoots and encouraged their elongation after a seven-week transition from a solid to a liquid medium [53,54]. 

Indirect morphogenesis: Meanwhile, in the case of indirect morphogenesis, the callus tissue is genetically unstable. The callus formed can help enhance *Chrysanthemum* species’ genetics by showing helpful genes or by incorporating new cultivars [53,54]. In addition, the in vitro shoot regeneration of *Chrysanthemum* cv. appears to depend on selecting a donor plant age that produces the most shoots per explant. For instance, leaf disk explants from 6-week-old donor plants (*Chrysanthemum* cv. *Shinma*) formed shoot buds 1 week earlier (than other donor plants). They produced the highest number of shoots per explant (7.2) in the medium, implying that donor plants that were either too young or too old had reduced regeneration efficiency [34]. 

## 3. Basal Medium for Regeneration

Tissue-cultured plants are grown on a synthetic medium containing all the necessary nutrients for rapid growth. Murashige and Skoog’s formulation (MS) can be used to grow various plants, resulting in enhanced growth [55]. According to Rahmy et al. [22], MS medium may be substituted with an artificial media, Grow More and a varied concentration of coconut water, to initiate the shoot regeneration of *Chrysanthemum* in vitro Plant tissue cultures can also be done in a liquid or semisolid media with a solidifier. Teixeira da Silva and Kulus, [51] developed a cost-effective method for the large-scale production of chrysanthemums cv. ‘Shuhou-no-Chikara’ in vitro, using various additions to a liquid-based medium. He also discovered that several alternative additions to the liquid-based medium are available such as coffee, Darjeeling tea, Japanese matcha, low and full-fat milk, Coca-Cola, and oolong tea, which inhibits plant development and reduces the concentration of leaf chlorophyll. Lee et al. [56] stated that agarose outperforms agar in shoot renderability promotion. However, Gelrite is the best gelling agent for accelerating shoot regeneration in *Chrysanthemum* cv. *Borami* and *Chrysanthemum* cv. *Vivid Scarlet* leaf explants than agar, agarose, or Phytagel [17]. Furthermore, psyllium husk can be used as a gelling agent in a culture medium because it is a sticky and mucilaginous substance [57,58]. In addition, [57,58] concerns the formation, proliferation, and long-term survival of in vitro shoots [18,46,59]. Earlier studies have also mentioned that all gelling agents tested had produced fewer shoots and roots than gellan gum and agar (bacto agar, oatmeal agar, Phytagel, potato dextrose agar, corn starch, and barley starch) [60]. Similarly, Gelrite produced more shoots per explant than agar, agarose, or Phytagel, but silver nitrate prevented the induction of shoots [51]. Similar results were also obtained when plants were cultured on media containing refined sucrose or table sugar, while extracts from *Stevia rebaudiana* (Bertoni), which is used as a substitute sweetener in food products, gave poorer results. Photoautotrophic micropropagation increased shoot mass, and the aeration of the culture vessel enhanced plantlet growth, which resulted in double plant density [51]. The physical state of other solidifier media with poor diffusion properties can impede nutrient flow, resulting in fewer shoots per explant, which is a plausible explanation for these observations [18,20]. Gelling agents and plant growth regulators may work together to boost shoot regeneration [17,18]. Plant tissue culture medium frequently uses sucrose as a carbon source to substitute the carbon, which plants usually fix via photosynthesis but cannot perform in vitro. Occasionally, organic substances such as amino acids and vitamins are added [4]. Market sugar was used instead of sucrose due to its similarity to sucrose, which contains 99.98% sucrose and 0.01% reducing sugar (compared to 96–97% sucrose and 0.7–1% reducing sugar in sucrose) [46,61]. Pant et al. [46] developed a modified culture media technique consisting of a full-strength MS medium supplemented with 0.5 mg/L BAP, a solidifying agent. Psyllium husk (Isabgol) also substituted the agar as a solidifying agent. In addition, market sugar (as a carbon source) and double distilled water were substituted by RO (reserve osmosis) water for making culture medium, resulting in a 6-fold reduction in production costs. Furthermore, it did not compromise on in vitro shoot development and was a cost-effective option. The starting material selection should be followed by a suitable sterilization process to successfully disinfect the explants without causing harm to them [60].

## 4. The Alternative Disinfection Methods of Culture Media

One of the most serious issues with micropropagation is microbial contamination, which results in the poor quality of plants and the destruction of beneficial stocks. Also, sterilized culture media may reduce the effectiveness of nutrients and growth regulators for plants [62]. Ornamental plants like chrysanthemums are usually cultivated in disinfected soils by cuttings or cultured in a medium to avoid contamination and maintain optimum conditions for homogeneous substances. Autoclaves are utilized to sterilize any cultured substances in many micropropagation laboratories. Typically, sterilizing is done by autoclave for the culture medium and culture vessels (plastic boxes, glass vessels, nylon bags) for 20 to 30 min at 121 °C and 15 psi. However, autoclaving can result in the production of decomposition products like phenolics and 5-(hydroxymethyl)-2-furaldehyde at a high cost to several micropropagation laboratories [63], and it can also result in the generation of decomposition products such as phenolics and 5-(hydroxymethyl)-2-furaldehyde [64]. 

Several recent techniques have been used to sterilize in vitro media without needing an autoclave and with a low-cost alternative, such as microponic culture system [20], sodium hypochlorite (NaClO) [39,65,66], hydrogen peroxide [67], and chlorine dioxide [68,69], metal nanoparticles [20,62,70,71,72]. On the other hand, chemical sterilization can effectively disinfect but is often harmful to explants and reduces propagation efficiency. As a result, nano colloids may be considered an easy and effective alternative to current methods for disinfection [70].

Creating a successful *Chrysanthemum* regeneration system allows the vital germplasm to be preserved through clonal propagation. This used a simple culture to vigorously multiply shoot tips on MS medium supplemented with 0.5 mg dm^−3^ BA + 0.1 mg dm^−3^ NAA to reduce the prevalence of seven viruses and viroids, including the chrysanthemum chlorotic mottle viroid (CChMVd), chrysanthemum stunt viroid (CSV), cucumber mosaic virus (CMV), chrysanthemum virus B (CVB), tobacco mosaic virus (TMV), tomato aspermy virus (TAV), and tomato spotted wilt virus (TSWV) [73].

### 4.1. Microponic Systems

**Figure 2:**
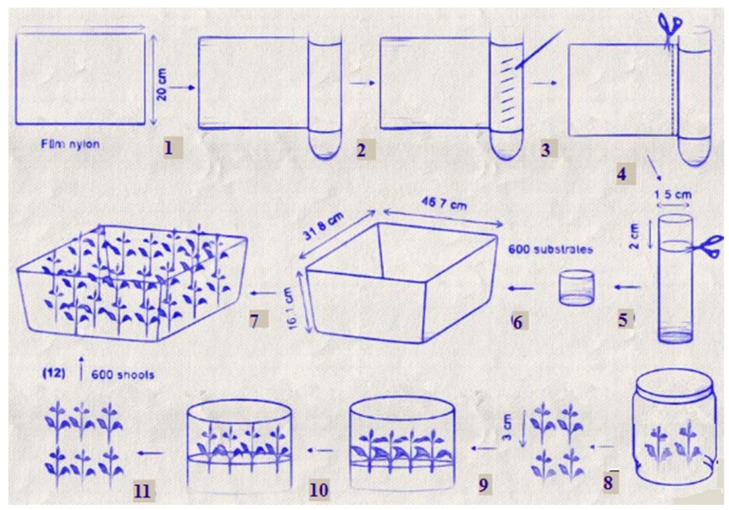
Microponic system diagram adapted from Tung et al., 2018 [20].

Microponic systems are reproduction systems that combine the benefits of micropropagation with hydroponics, which reduces the disadvantages of micropropagation systems of contamination, resource consumption, and the requirement for large spaces [20,74]. The pioneers of this method were [75,76], who used the nutrient film technique and a miniature pump to circulate medium through Rockwool (Figure 2). In addition, the culture conditions such as improved fresh and dry weight, rate of photosynthesis, leaf size and number, and stomatal density (temperature, CO_2_, humidity, pH, and electrical conductivity) are controlled. Tung et al. [20,77] concluded with a distinction between the microponic (MO) and micropropagation (MR) systems. In a microponic system, the volume of the container is 16.1 cm × 31.8 cm × 45.7 cm; the content of the medium = ½ MS, without sugar, pH 5.8); the substrate used is Nylon film, and there is no autoclave. While in the micropropagation system, the volume of the container is a 500 mL glass bottle; the content of the medium = ½ MS, 30 gL^−1^ sucrose and pH 5.8; the substrate is 8 gL^−1^ agar, and the method of sterilization is autoclave (121 °C at 1 atm for 30 min) [20]. The length of the chrysanthemum shoots utilized was 3 cm, grown on half-strength sugar-free liquid MS media under a 70/30% red/blue LED setup with 7.5 ppm silver nanoparticles. Chrysanthemums grown in a microponic system began flowering after 15 weeks, which was a week earlier than those grown in vitro, and branches derived from microponic culture may blossom one week earlier than micropropagation-derived branches. 

### 4.2. Essential Oils and Chemical Compounds in Tissue Culture Media

Without an autoclave, plant tissue culture material can be sterilized by chemical disinfectants and essential oils (EOs) derived from medicinal plants [25,78]. For example, EOs of trees of betel, cinnamon, clove, holy basil, lemon, lavender, turmeric, and tea (at varying concentrations: 0.9 mL dm^−3^–12.6 mL dm^−3^) might also 100% sterilize the medium, which is equivalent to autoclaving [51]. Similarly, the subsequent compounds can be utilized as a sterilant: 2% iodine + 2% merbromin solution, 2.4% potassium iodide, 10% povidone–iodine, 6% sodium hypochlorite, or 0.1% thimerosal at 1.8 mL dm^−3^; these could also achieve 100% medium sterilization as well. This method offers a less expensive substitute in laboratories that do not have any autoclaves, although contradictions surface if EOs would be less expensive than autoclaving. [79] determined that chemicals like chlorine, calcium hypochlorite, sodium hypochlorite, hydrogen peroxide, methylchloroisothiazolinone, magnesium nitrate, magnesium chloride, sodium benzoate, and potassium sorbate ought to be present in the culture medium to avoid contamination. According to Liu et al. [39], the best sterilization approach for *Chrysanthemum morifolium* ‘ziyan’ was applying 2% NaClO for 6 min and for *Chrysanthemum morifolium* ‘niu 9722′ was applying 2% NaClO for 8 min. These chemicals frequently harm plant tissue (especially in chimeras, especially those of the “Variegata” type”) or are ineffective at eliminating fungal and endophytic bacterial contamination [51]. 

### 4.3. Nanomaterials in Tissue Culture Media

The interactions between nanomaterials and plant growth have recently piqued the interest of experts worldwide [20,62,71,72,80]. Silver nanoparticles (AgNPs) have previously been shown to prevent microbial infection and the effects of ethylene in micropropagation [20,62,71,72,81,82,83]. Silver and copper nano colloids also have antibacterial, antifungal, and antiviral properties, but they are less toxic and need not be rinsed with sterile water. They can also destroy endophytes by entering the cell through plasmodesmata [84]. In addition, non-autoclaved media can be utilized for cultural purposes due to lower micropropagation costs and lower power consumption (attributed to the absence of autoclaving). Furthermore, AgNPs are critical for improving the growth and development of plants (shoot as well as root length and leaf area), with a better synthesis of chlorophyll and oxidative enzymes, enhancing the carbohydrate and protein content of chrysanthemum. AgNPs, when added to a microponic medium, can enhance plant growth and development while reducing microbial contamination [20,62,70,82,85]. Copper (Cu), gold (AU), and silver (Ag) nano colloids can also be used to eliminate fungal and bacterial contamination in *Chrysanthemum* in vitro cultures [83]. The nano colloids showed encouraging antibacterial and antifungal efficacy, even at lower concentrations for a brief period of disinfection. In addition, there was no apparent injury to plant tissue. According to a study conducted by Tung et al. [20] and Tymoszuk and Miler [83], the probability of microbial infection was determined by adding different AgNP and AuNP concentrations to micropropagation and microponic system. In comparison to silver nanoparticles, gold nanoparticles are less toxic to in vitro isolated plant explants. Adventitious root regeneration in *Chrysanthemum* was restricted after exposure to AgNPs at concentrations of 10 and 30 ppm. The in vitro rhizogenesis of these species should not involve silver nanoparticles. Even so, chrysanthemums exposed to AuNPs (at 10 and 30 ppm) had roots that were significantly larger in diameter. However, silver nanoparticles at concentrations of 50 and 100 ppm restrict the development of adventitious branches in *Chrysanthemum* [83]. The optimum concentration of AgNP was found to be 10 ppm for reducing microbial content among the tested concentrations, but this concentration also inhibited plant growth, causing leaf distortion and, finally, death. Furthermore, the plant roots became brown and eventually perished. In comparison to 5 ppm AgNP, AgNP at a concentration of 7.5 ppm was the most effective at lowering the levels of several bacteria, including Xanthomonas sp., Enterobacter sp., Bacillus sp., *Pseudomonas* sp. (<1 CFU/mL), and appropriately controlled *Alternaria* sp. (diminished 2500-fold). AgNP at 7.5 ppm also reduced the growth of *Corynebacterium* sp., *Agrobacterium* sp., and *Aspergillus niger* by 10-fold compared to controls (3-fold). Some species (*Fusarium* sp. and *Arthrobacter* sp.) were unaffected by AgNP, while there was optimal development of *Chrysanthemum*, and plants cultured in a medium supplemented with 7.5 ppm AgNPs showed higher chlorophyll contents.

The sterilizing effects of silver nanoparticles (AgNP) on *Chrysanthemum morifolium* (Ramat.) cv. “Jimba” explants growth and culture media were studied by Tung et al. [62]. Compared to the autoclaved (Au M) system, plantlets from the non-sterilized MS medium (No M) system adapted to the greenhouse faster. *Chrysanthemum* plantlets grown in the non-sterilized media system had higher superoxide dismutase and ascorbate peroxidase efficacies than in the autoclaved medium. The No M1 and No M2 plantlets (in two large plastic containers, each with a different size) had reached their developmental stages one week earlier than the AuM system (flower buds and blooming period). 

### 4.4. Reintroduction of Arbuscular Mycorrhizal Fungi (AMF) in Tissue Culture Media

Reintroducing arbuscular mycorrhizal fungi (AMF) into sterilized areas is an alternative technique to eradicate harmful pathogens and beneficial microbes while reaping the benefits of AMF–plant root symbiosis [51]. Since microbial inoculum is essential for developing a strong root system [86], improved growth [87], increased absorption of nutrients and water [11,88], and improved host root resistance to soil-borne diseases [89] and drought stress [90]. Micropropagated plantlets can have proper growth attributed to AMF if they are inoculated with it. This will be reflected in improved plant survival and development after field transplant. AM fungi are an integral part of *Chrysanthemum* micropropagation, which will enhance the uptake of nutrients whether they are given with macronutrients (N, P, K, Ca, and Mg) or micronutrients (Fe, Cu, Zn, and Mn) in both shoots and roots of plantlets that have been inoculated with a mixture of three strains of AMF including, *Acaulospora laevis, Acaulospora scrobiculata,* and *Glomus fasciculatum* [11].

Mycorrhizal inoculation from scarcely soluble sources, such as rock phosphate, is made available for the plant to obtain phosphorous content. The enhanced phosphorus uptake may be due to the increased physical interaction between phosphate particles and the hyphal network between roots and these particles. AMF has been shown to facilitate nodulation and nitrogen fixation in legumes. Mycorrhizal and nodule symbioses often synergistically affect mineral nutrition, infection rates, and plant growth. The enhanced phosphorus uptake by AMF symbionts is beneficial for the nitrogenase enzymatic activity in bacterial symbionts, leading to higher nitrogen fixation and, consequently, stimulating the development of root and mycorrhiza. Further, increased micronutrient uptake may be associated with increased macronutrient cation mobilization in the rhizosphere via secretions of AMF. The blend of specific chelating siderophores by strains of AMF may contribute to enhanced iron absorption [91].

### 4.5. High-Energy Photons and Electrons

Industrial sterilization, which uses high-energy electrons and photons, is one application of ionizing radiation [92]. According to Miler et al. [14], high-energy electrons are more effective at disinfection than high-energy photons. The effect of radiotherapy on the percentage of sterile *Chrysanthemum* explants was noted, and as the radiation dose increased (gradually, from 55% in 5 Gy photons to 70% in 15 Gy photons), the percentage of sterile ovaries explants also increased. In parallel to the control, the increased level of (complete) infertility was caused by ovaries receiving 10 Gy of high-energy electrons [14]. 

## 5. Protocols for Cloning and Large-Scale Plant Production of *Chrysanthemum*

Plant growth regulators or phytohormones, such as cytokinins, gibberellins, auxins, and abscisic acid, as well as their analogs and inhibitors, are essential to controlling the type of growth during the proliferation stage. Growth regulators can have distinct effects on various cultivars due to genotypic changes in their capacity to absorb and metabolize the medium’s growth regulators [93]. Variations in growth conditions and explant source age, genetic variations between the genotypes used, or morphogenetic response variations in vitro can contribute to these variations [35]. A summary of recent studies focused on the optimal cultured medium used for in vitro for chrysanthemum proliferation is listed in Table 1.

### 5.1. Optimization of Phytohormones on the Shoot, Callus, Somatic Embryo, and Root Induction

*Chrysanthemum* shoot regeneration is induced by the medium’s type and concentration of growth regulators. Lower concentrations fail to promote shoot bud regeneration, while higher concentrations have an inhibitory effect as the plant itself can produce hormones [23]. The residual effects of hormones accumulating in cultured explants and the application of plant growth regulators (PGRs) could explain why the number of shoot buds/explants was reduced at higher combined concentrations. Furthermore, Waseem et al. [36] found that augmenting MS medium with increased benzyl amino purine (BAP) reduced recovery. It is due to endogenous cytokinins such as elevated concentrations of 6-benzyl adenine (BA), thidiazuron (TDZ), and BAP used that may have caused adverse consequences and reversed the growth process [101]. In contrast to the common assumption, high levels of auxin cause rhizogenesis, whereas high levels of cytokinin cause ridge formation. Some strains require higher concentrations of cytokinin than auxin or auxin at a higher concentration than cytokinin and comparable amounts of auxin and cytokinin [102]. Further, cytokinins play an important role in shoot regeneration in plant tissue culture, and BAP is one of the most potent cytokinins for inducing shoot regeneration [103]. For example, high levels of cytokinins induce explant germination (6-benzylamino purine (BA), zeatin, kinetin, and 6-(γ,γ-dimethylallylamino) purine) [104]. Cytokinins other than auxin were used for leaf explants to promote efficient direct organogenesis in *Chrysanthemum* [105,106]. Similarly, BAP is more effective than kinetin (Kin) in strengthening shoot amplification in *Chrysanthemum* and other plant species, as mentioned in [107,108,109]. The utilization of different concentrations of cytokinin and auxin in tissue culture of (Pyrethrum) *Chrysanthemum cinerariaefolium* was examined for the first time by Lindiro [110], which investigated nodal explants in MS medium treated with various amounts of cytokinins, 2-isopentyl adenine (2iP), benzylamino purine (BAP), kinetin (KIN), thidiazuron (TDZ), and cysteine. According to these findings, BAP was superior in propagating axillary shoots, with 5 M BAP yielding the highest average shoot length and 40 M BAP yielding the highest average number of shoots. The superiority of BAP for inducing axillary buds in *C. morifolium* was demonstrated by Pant et al. [46]. The ability of low BAP levels itself stimulates shoot growth and proliferation, and callus development in the presence of auxin reflects high endogenous hormone levels in the mother plant [46]. The endogenous content of cytokinins found in leaves of *Chrysanthemum* was much lower to stimulate shoot regeneration. One of the biological functions of KIN, a cytokinin, is to inhibit apical dominance, increase lateral shoot growth, and create vegetative shoots [100]. It also helps discover and enlarge blood vessels that carry phloem and xylem, inhibiting chlorophyll breakdown, promoting cell division, and improving nucleic acid production [111,112]. Additionally, ClO2 was investigated as a growth stimulant for chrysanthemum tissue culture without using any other known PGRs in a recent study by Tian et al. [69]. It was observed that 10 μg·L^−1^ ClO2 caused *Chrysanthemum* regeneration in a single step. This shows that a microgram-grade concentration of ClO2 may stimulate the accumulation of endogenous auxin in *Chrysanthemum*, further encouraging roots and growth. The regenerates formed in a single step and were transplantable within three weeks of culture. The transplantation success rate was 100%.

#### 5.1.1. Shoot Induction

Previous experiments on shoot regeneration have been done in *Chrysanthemum* to study the effects of silver nitrate, which is an ethylene inhibitor [18,34,56,113]. Naing et al. [34] showed that ethylene inhibitors adversely affected shoot growth in the leaf explants of *Chrysanthemum* cultivars. The magnitude of these adverse effects depends on the type and amount of ethylene inhibitor used, such as AVG, silver thiosulfate, and silver nitrate. The addition of 10–20 mM silver nitrate promoted shoot regeneration in *Chrysanthemum* explants grown in an environment richer in cytokinins than auxins. Similarly, the average number of shoots per explant was not affected by silver nitrate concentrations above optimal levels, indicating a strong interaction between silver nitrate and cytokinins in Chrysanthemums [113]. Adding 1 mM silver nitrate increased the number of shoots per explant when *Chrysanthemum* explants were cultured in a medium containing equal amounts of cytokinin and auxin, as was observed by Lee et al. [56]. Silver nitrate did not interfere with shoot regeneration, even at 10 mM. In addition, shoot development occurs at silver nitrate concentrations of up to 100 mM. On the other hand, *Naing* et al. [34] showed that explants cultured in a medium containing 1 mM silver nitrate markedly hampered shoot regeneration, whereas 25 mM silver nitrate completely prevented shoot regeneration. Furthermore, according to Naing [18], shoot regeneration could be accelerated by adding silver nitrate to a medium with high doses of cytokinin, well known for promoting the in vitro production of ethylene. It is thought that silver nitrate increases ethylene absorption and improves shoot regeneration. Furthermore, cytokinins and silver nitrate have a significant interaction in *Chrysanthemum*, since cytokinins are known to stimulate ethylene synthesis in vitro. In addition, silver nitrate is known to absorb ethylene and enhance shoot regeneration. A possible reason for this discrepancy is the use of higher concentrations of cytokinins in the study conducted by XiaoHan et al. [113]. Differences in endogenous ethylene levels between plant genotypes are the source of the above-mentioned discrepancies [34]. Silver nitrate and other ethylene inhibitors may enhance embryogenesis in species with high endogenous ethylene levels while suppressing development in species with lower endogenous ethylene levels [114]. The stunted and dwarfed in vitro-generated shoots were unsuitable for subculture and in vitro rooting. Consequently, before in vitro roots can be produced, these micro shoots need to grow and develop sufficiently. Previous research by Jerzy et al. [94] solves the issue of *Chrysanthemum* branches grown from floral explants not growing to the length that makes it easy to separate them from the explant by adding kinetin to the medium. Consequently, the length of the shoots that emerged from ligulate florets on the medium containing 1 mg dm^−3^ KIN was nearly three times greater than the length of the explants present throughout the culture period on the medium with BA and IAA. A study by Jahan et al. [23] experimented on *Chrysanthemum morifolium* and showed that multiple shoots were sub-cultured in the BAP-enriched MS basal media with 125 mg/L urea, and the length grew to a size that was sufficient in three to four weeks.

#### 5.1.2. Callus Induction

In callus induction [60], auxin alone may cause the formation of calluses on explants if the auxin balance in the explant is sufficient or if the cytokine concentration is extremely low or nonexistent. Auxin can enhance adventitious root development, cell elongation, and cell division, according to Pierik [115]. Due to comprehensive and successful interventions in the cell cycle and cell division, both cytokinin and auxin are effective in forming calluses and somatic embryogenesis in *Chrysanthemum* species [116]. Furthermore, 2,4-D concentrations between 0.1 and 2.0 mg L^−1^ are required for embryogenic callus development from leaf and nodal explants [117]. It has been demonstrated that 2,4-D at a concentration of 2 mg L^−1^ is a relevant medium for inducing callus in *Chrysanthemum* plants [118]. Siregar [119] discovered that, when TDZ was added to the planting media at concentrations greater than 0.25 mg L^−1^, it prevented leaf callus explants from producing shoots and reducing their length. The flower tissue of *Chrysanthemum* cv. ‘Shuhou-no-Chikara’ was found to have organogenesis for the first time due to the five PGRs, adenine sulfate (Ads), picloram, N6 –[2-isopentenyl] adenine (2iP), phloroglucinol (PG), and coconut water (CW). Callus was formed by picloram, 2iP, thidiazuron (TDZ), and 2,4-dichlorophenoxyacetic acid (2,4-D), and Ads, BA, and KIN formed shoots. Shoots and callus were formed by CW, and PG; indole-3-acetic acid (IAA) and indole-3-butyric acid (IBA) formed the roots [120]. The callus was propagated on an MS medium containing 3% sucrose and 0.8% agar with l mg/L BAP for culturing leaf explants of *Chrysanthemum*. According to [121], using the right amount of auxin concentration in in vitro culture can slow down morphogenesis while accelerating the growth of calluses. The rate of callus formation increased with increasing concentrations of 2,4-D (2–4 mg L^−1^), then declined with higher concentrations. It was observed that a combination of 6-BA and NAA in different concentrations was best for callus induction, stem proliferation, and having a 100% survival rate on *Chrysanthemum morifolium* cv. “niu9722” and *C. morifolium* cv. “ziyan” [39]. In contrast, in the culture of chrysanthemum callus, different concentrations of TDZ prevented the formation of shoots [95]. Rivai and Helmanto [122] explored how 2,4-dichlorophenoxy acetic acid (2,4-D) produced calluses in chrysanthemum explants like leaves, hypocotyls, cotyledons, stems, zygotic embryos, and other plant parts cultured in auxin-rich medium. The ideal treatment for inducing callus from leaves was MS medium enriched with 3 mg/L of 2,4-D, and MS medium enriched with l or 2 mg/L of 2,4-D was the best method to induce callus from internodes. The callus became brownish when the 2,4-D amount was increased [123]. 

#### 5.1.3. Somatic Embryogenesis Induction

The most economically significant *Chrysanthemum* cultivars and plant regeneration can be improved with a different but equally efficient somatic embryogenesis process. This process is a distinct developmental pathway characterized by the dedifferentiation of cells; cell division induction; cell division stimulation; and the reprogramming of cell metabolism, physiology, and gene expression patterns [124]. In tissue cultures of *Chrysanthemum*, somatic embryogenesis can be done either directly from the epidermal cells of explants [53] or indirectly through an intermediary callus [125]. Somatic embryogenesis is a complex process that depends on a wide range of factors, such as plant genotype, culture media composition, the type and age of the explants, and various types and concentrations of phytohormones [98,99]. PGRs of the right type and concentration, coupled in diverse ways, can induce somatic embryogenesis [98,126]. Barakat et al. [50] showed that increased BAP and NAA concentrations in the medium enhanced the somatic embryogenesis of *Chrysanthemum morifolium* to produce somatic embryos. Mani and Senthil established a methodology for the proliferation of *Chrysanthemum cinerariaefolium* [127] via somatic embryogenesis, utilizing 100% callus induction from leaf explant on MS media containing 1.5 mg/L of 2,4-D and petal explant on MS medium containing 2.0 mg/L of 2,4-D. For somatic embryos, The strongest friable calli for somatic embryos were tested in MS medium that had BAP (0.1 mg/L) treatment. The regenerated plantlets were elongated and planted on MS media supplemented with 0.1 mg/L of BAP and 2.0 mg/L of KIN. Leaf explants demonstrated the highest response rates of any explant type, with rates of embryogenesis at 97.9% and 35.1%, respectively, for petiole and internode stem explants. A study by Keresa [126] stated that both plant growth regulator combinations (BA and GA3) significantly influenced embryogenesis. Leaf explants demonstrated the most response rates of any explant type, whereas petiole and internode stem explants had the highest rate of embryogenesis, 97.9% and 35.1%, respectively. The highest conversion rate (53.8%) from somatic embryos to plantlets was seen in petiole explants. Therefore, petiole explants were the effective option for *Chrysanthemum* cv. *Palisade* White for plant development through somatic embryogenesis [126]. MS medium was shown to be more effective than half-strength MS medium in enhancing the proliferation of somatic embryos [128]. “Petal” explants from two rare *Chrysanthemum* cultivars, ‘Euro’ and ‘Baeksun’, were cultured in vitro by Naing et al. and Kim and Naing [18,128] to stimulate primary and secondary somatic embryogenesis. For the ‘Euro’ variety (42 embryos/explant following 5 weeks of culture), MS media enhanced with 2 mg dm^−3^ of 2,4-D and 2 mg dm^−3^ of KIN was ideal, but for the ‘Baeksun’ variety (56.3 embryos/explant following five weeks of culture), 1 mg dm^−3^ of 2,4-D and 3 mg dm^−3^ BA were sufficient. The optimum parameters for the embryo regeneration of the ‘Cool Time’ floral explant were determined by Tymoszuk et al. [129]. The best results were observed when transversely cut-in-half ligulate florets were cultured into MS medium with 1 mg dm^−3^ of KIN and 4 mg dm^−3^ of 2,4-D, wherein 5.7 embryos/explant and 85% explants were regenerated. Ray florets of the *Chrysanthemum* cv. ‘Purnima’ has been utilized to standardize an effective direct somatic embryogenesis process. Somatic embryos were produced immediately on the surface of the explant, escaping the callus stage [123]. A modified MS medium containing 15.45 mM NH^4+^, 38.95 mM NO_3_, 25.12 mM K^+^, 7.5 mM Cl, 3.75 mM Ca^2+^, 1.87 mM Mg^2+^, 1.87 mM SO4_2_, and 1.62 mM H_2_PO_4_ allowed for the maximum callogenesis ratio (100%), embryogenesis ratio (100%), and somatic embryo quantity (12.11). In addition, MS media enriched with different combinations of 2,4-D/BAP were used for *Chrysanthemum* callus generation from the cut end of the leaf disc, and it could generate successfully. In two weeks, the surface of the whole explant was covered in calli. MS medium supplemented with 2 mg L^−1^ of 2,4-D, and 2 mg L^−1^ of BAP gave the highest CR, ER, and SEN values [98]. 

#### 5.1.4. Root Induction

The root initiation stage, also known as in vitro rooting, ensures the survival of cloned micro cuttings and speeds up the rooting process. When auxin is present, the majority of the roots are stimulated. In general, a high percentage of rhizogenesis was enhanced by indole acetic acid (IAA), 6-benzyladenine (IBA), isopentyl adenine (2iP), and α-naphthalenacetic acid (NAA). Treatment with cytokinin may be less effective if apical dominance is present because auxins are naturally produced by apical buds [130]. In general, auxins promote cell division and elongation and encourage root formation. The application of growth regulators, resulting in an excess in the average number of roots while increasing the concentration of growth regulators, has negative effects. Treatment with half the salt content was more effective because the carbohydrate-to-nitrogen ratio was increased. It is generally accepted that increasing the sugar-to-nitrogen ratio improves rooting [131]. Roest and Bokelmann [131] published the first tissue culture study of *Chrysanthemum cinerariaefolium* detailing the explantation of floral discs for the micropropagation *of Pyrethrum*. Explants were grown in MS medium supplemented with 10 mM (IAA), (NAA), and 0.1 mM (IBA). After eight weeks, the stimulation of these growth regulators produced plantlets, and proper transplantation was performed into non-sterile soil, which allowed the roots to grow. Obukosia et al. [118] reported that NAA was superior to IBA for rooting *Pyrethrum* micro shoots. The results were inconsistent with the outcomes of Waseem et al. [36], that BAP at 0.2 mg/L was superior to NAA for root induction from *Pyrethrum* micro shoots. Keresa et al. [126] examined that IBA (0.5 mg/L) produced more roots per shoot, while IAA (2 mg/L) generated longer roots. Lindiro [110] showed that 10 μM of IBA resulted in the highest number of roots per explant, the highest average root length, and the successful establishment of regenerated plantlets in the greenhouse.

Similarly, on MS medium supplemented with IBA (2.0 mg/L), regenerated shoots elongated and developed roots before being adapted and planted in the soil, although quantitative and qualitative features were completely inferior to TDZ [132]. Furthermore, in the previous studies [19], IAA had little effect on root induction. In contrast, IBA had the most positive effects on root induction and elongation. Similarly, Naing et al. and Jahan et al. [23,34] noted that IBA was the best auxin for adventitious root initiation, surpassing IAA and NAA. On the other hand, the most efficient medium for root regeneration medium was SH [133], and the optimal situation for the number of roots per explant (4.3) and root length is half the intensity of SH (1/2 SH) (31.4 mm) [19]. In contrast, Fu-Yun [42] observed that, on half-strength MS medium supplemented with 0.1 mg/L of NAA, the rooting rate for *Chrysanthemum nankingense* was 100%. The transplanted plants grew properly due to an average rooting coefficient of 15.8 and had a survival rate of 100%. Similarly, Wang et al. [44] reported that the optimum rooting media for ‘Breeze Ivory’ was a composition of (1/2MS + 7.0 g/L of agar + 0.1 mg/Lof NAA + 30 g/L of sucrose), with a rooting rate of 100%, and an average of 12.6 roots per plant, along with a 98% survival rate in river sand medium. Imtiaz et al. [134] found that ½ MS medium was optimal for the rooting of *Chrysanthemum*. Furthermore, Verma’s [135] explants (axillary buds) of *Chrysanthemum morifolium* containing well-differentiated micro shoots were placed on MS medium supplemented with 0.5 mg/L of NAA, and the cultures generated the highest roots (88.66%) in parallel with 1.0 mg/L of NAA.

### 5.2. Plant Growth Regulators in the Best Combination

The growth regulators’ interaction determines the speed and direction of the development of culture supplemented to the medium and those generated endogenously by plant cells (Gunawan et al. [136]). It has been suggested that two important factors, the chemical base and its side-chain groups, could explain the variations in the effectiveness of plant growth regulators on plant development [137]. Different plant or shoot regeneration responses are produced when different culture medium compositions are used; the developmental stages should be considered when choosing the culture medium with various hormone combinations [42]. A particular cytokine called BAP is frequently combined with NAA (auxin) to enhance the growth of plant shoots. BAP has been found to boost the synthesis of tissues of natural hormones like zeatin and is assimilated by plant tissues more rapidly than other synthetic growth regulators [110,138]. On the contrary, it has also been demonstrated that BAP, in combination with IAA [47] and GA3 [126], causes *C. morifolium* to induce the largest shoots. Auxin and cytokinin, the two plant growth hormones, are commonly used to stimulate morphogenetic plants [95]. According to Ilahi [139], MS media with 0.5 mg L^−1^ of NAA and 0.5 mg L^−1^ of BAP was the most optimum medium for producing calli in *Chrysanthemum* explants taken from internodes. In addition, [42] explored the observation that early bud induction was observed after five days of growth on a half-strength MS medium boosted with BA (0.5 mg/L) and NAA (0.1 mg/L). Furthermore, Waseem et al. [47] reported that adding low concentrations of IAA (0.1 and 0.2 mg/L) and moderate concentrations (1.0 and 2.0 mg/L) of BAP to MS medium improved *Chrysanthemum* plantlet regeneration utilizing nodal segments. Shoot tips from *Dendranthema × grandiflora* (Ramat.) Kitamura cv. Palisade White was cultivated on MS media with combinations of (BA, GA3) or (BA, KIN, and IAA). The combination of BA at 0.1 mg/L and GA3 at 0.5 mg/L led to a proliferation rate of 3.2 new micro shoots per inoculated plant [126]. Furthermore, Wang et al. [44] suggested the ideal breeding medium for *Chrysanthemum* cv. ‘Breeze Ivory’ was a composition of MS medium with 6-BA (1.0 mg/L) + NAA (0.1 mg/L) + sucrose (30 g/L) + agar (7.0 g/L) with a multiplication coefficient of 12.1. In a study by Naing et al. [128], a 5-week culture gave the highest average number of embryos per explant (5.97) when *Chrysanthemum* cv. Euro leaf explants were cultured on MS medium supplemented with 2.0 mg/L of kinetin and 2.0 mg/L of 2,4-dichlorophenoxyacetic acid. Tymoszuk and Zalewska [140] also showed that adventitious buds could be regenerated from “Cool Time” ligulate florets as long as the medium containing appropriate amounts of BA and NAA. Medium supplemented with 2.0 mg dm^−3^ of BA and 0.3 mg dm^−3^ of NAA and media with (2.0–3.0) mg dm^−3^ of BA and 0.5 mg dm−3 of NAA resulted in the majority of shoots per flower explant (8.89–36.09) being regenerative. In vitro studies were performed on cultured petal explants of his cultivars of *Chrysanthemum*, i.e., “Resomee Splendid” and “Reagan Elite Salmon”. In MS medium, combinations of 3.0 mg/L of BAP with 0.5 mg/L of NAA and 4.5 mg/L of BAP with 1.0 mg/L of NAA, the maximal shoot induction of both cultivars (93.33% and 73.33%, respectively) was achieved by [132]. Random shoots were also reported to occur indirectly in ‘Resomee Splendid’ explants, whereas in ‘Reagan Elite Salmon’, either a combination of (3.0 mg/L of BAP + 0.5 mg/L of NAA) or medium supplemented with (4.5 mg/L of BAP + 0.5 mg/L of NAA) directly, shoot formation was observed [132]. In both cultivars, a mix of TDZ and NAA was developed with indirect shoot development. Naing et al. [18] suggested that, for the successful genetic transformation of *Chrysanthemum* cultivars, in the presence of 1 mg dm^−3^ of BA and 2 mg dm^−3^ of NAA, high levels of auxin were required for shoot regeneration; leaf segments of ‘Vivid Scarlet’ could produce 12.3 shoots per explant. The suggested medium for chrysanthemums for shoot regeneration of ‘Biarizte’, ‘Yellow Biarizte’, ‘Storika’, ‘Pinkgin’, ‘Linker Pink’, ‘Dark Linker Salmon’, and ‘Bari’ from ligulate florets comprised 1.5 mg/L of BAP and 0.5 mg/L of NAA; ‘PKV Shubhra’ required 2 mg/L of BAP and 1.5 mg/L of IAA [141]; ‘Shiroyamate’ required 3 mg/L of BAP and 10 mg/L of NAA [142]; and ‘Breeze White’ and ‘Capitola’ required 3 mg/L of BAP and 0.5 mg/L of IAA. The medium containing BAP was swapped out with 1 mg/L of KIN, and in “Capitola,” twice the number of explants underwent shoot regeneration, accounting for more than half of the total [94]. Similarly, for the efficient regeneration of *Chrysanthemum morifolium* (Ramat.), both NAA and BAP at 0.5 mg L^−1^ showed the earliest shoot induction (30 days) and highest number of shoots (5 shoots), with the longest shoot length (2.88 cm); similarly, NAA (1.0 mg L^−1^) and BAP (0.5 mg L^−1^) generated the heaviest callus [95]. On the other hand, 2iP (0.5 mg L^−1^) combined with BAP (2.0 mg L^−1^) could produce shorter shoots (1.88 cm) and only one shoot, whereas BAP (0.5 mg L^−1^) added without 2iP could produce shorter shoots (1 cm) with a slower shoot initiation (39 days) [95]. An addition of 0.5 mg.L^−1^ of NAA supplemented with 2.0 mg.L^−1^ of BA to MS media gave the best results in a single-node culture of *Chrysanthemum,* in terms of mean shoot number (86.6%) and mean shoot height (3.7 cm) [143]. Naing et al. [34] suggested that different combinations of BA and IAA concentrations did not affect shoot regeneration in cv. Shinma. Compared with the applications, the medium mixed with 1.0 mg L^−1^ of IAA and 0.5 mg L^−1^ of BA resulted in successful shoot development. However, the rate of shoot regeneration and the number of shoots per explant were low. In contrast, NAA at 0.5 mg L^−1^ in combination with 0.5 mg L^−1^ of BA was considered optimal for cv. Shinma for in vitro shoot regeneration. Observations by Imtiaz et al. and Zafarullah et al. [134,144] suggested that a lower NAA concentration with 6-BA concentration gave more shoot buds/explant than a higher concentration in combination supplemented in an MS medium. KIN and IBA are involved in preventing chlorophyll, protein degradation, and promoting photosynthetic enzymes, thus having a positive effect on increasing shoot number and length, both of which elevate cell size and induce cell division with formal differentiation. Alsoufi et al. [100] demonstrated that the interaction between 4 mg·L^−1^ of KIN and 0.6 mg·L^−1^ of IBA had a significant positive effect on the average number of shoots developed on the individual nodes of *Chrysanthemum* plants and the average shoot length (cm).

### 5.3. Optimization of Light Conditions

High shoot regeneration was attained during the dark incubation period, possibly due to an accumulation of auxin. Above the optimal concentration, auxin aggregation is more likely to prevent shoot regeneration [102]. The incubation period in the darkness of 10 days produced greater shoot regeneration in most cases compared to other durations (0, 20, 30 days), according to Naing et al. [18]. In addition, explants housed in darkness for a week had the greatest degree of shoot regenerability, followed by explants incubated in light (control) [34]. Meanwhile, darkness lasting longer than seven days exhibited inhibiting effects, and longer periods of darkness (in sequence 4 > 3 > 2 weeks) had greater inhibiting effects [18,34]. Endogenous auxins are supposed to accumulate when explants are incubated in the dark; however, excessive auxin aggregation prevents shoot regeneration [145]. In contrast to earlier research, [102] found that *Chrysanthemum* leaf ex- plants benefited most from a 12- to 18-day dark treatment for optimal shoot induction. Variations in explant types, genotypes, and plant growth regulators could bring these changes. On the contrary, a study by Teixeirada Silva and Kulus [51] found that explants of *Chrysanthemum* cv. “Shuhou-no-Chikara” responded uniformly to various plant growth regulators in light and dark circumstances. This is true for both disc and ray florets.

## 6. Irradiation Treatment In Vitro

Changing the color of the flowers is one of the most important breeding goals. Classical mutation breeding is viable for commercial plant breeders because it does not require advanced molecular laboratories with high-tech instruments or expert technicians with genetic engineering degrees. It is also ubiquitous and does not need an in-depth comprehension of gene sequences, structures, and functions of genes [146]. A remarkable number of novel chrysanthemum varieties are submitted to the Community Plant Variety Office (CPVO) each year. This European organization is equivalent to the parallel office for protecting breeders’ property [147]. Greenhouse tests confirm the nominated varieties’ uniqueness, uniformity, and stability (so-called DUS tests). In the CPVO department, cuttings of a given cultivar are grown at the CPVO department in a certain number of cuttings (20 for chrysanthemums) are grown, and their novelty is verified based on the assessment of their external traits [148].

Many studies have been published on the mutation breeding of *Chrysanthemum,* using physical and chemical mutagens. Among the physical mutagens, heavy-ion beams and X-rays or gamma rays are less harmful to the environment than chemical mutagens due to the production of toxic chemical waste and chemical agents. The most common chemical mutagen is ethyl methanesulfonate (EMS), but pingyangmycin (PYM) has also gained similar interest; it is an antibiotic used in cancer treatment [149]. Furthermore, due to their high availability, microwaves (MW) are a more effective and cheaper alternative to induce plant mutation than using less available gamma or X-ray radiation or user-harmful chemical mutagens in plant mutation breeding works. Water molecules in all living cells can absorb this radiation [150].

MW is a form of electromagnetic radiation (EM) with frequencies between 300 MHz to 300 GHz and wavelengths between 1 m to 1 mm [151]. Following the use of gamma radiation to induce mutations in a purple-flowering cultivar, *Chrysanthemum grandiflorum* (Ramat./Kitam.) emerged with three new phenotypes: light purple (77B), silver–purple (RHSCC code: 77C) and claret gold (60C), which was already confirmed by Zalewska et al. [26]. Miler and Kulus [97] studied the effect of microwave radiation on in vitro regeneration and the efficacy of acclimatization effectiveness, and genetic and phenotypic differences in *Chrysanthemum* ‘Alchemist’ were also observed. When using an MW radiation source with a power of 800 W·cm^−2^ and a frequency of 2.45 GHz, shoot production was adversely affected when exposed to long-term use. However, it did not impact the rooting and acclimation processes, both of which were completed.

Moreover, the propagation of inflorescences with larger diameters (21.5%), different shapes, and flower bud color was extended by four days with the longest treatment of MW. Similarly, comparable results were obtained in *Chrysanthemum* ‘Lalima’ irradiated with 0.5 Gy gamma rays [152]. Increasing the duration between the appearance of buds and the onset of flowering is beneficial, as it may improve the quality of the plant after harvest. These findings can potentially expand the use of microwaves as an inexpensive and widely available source of variation that is accepted by society. Commonly used explants in the mutation breeding of *Chrysanthemum* are: fragments of leaves and internodes, pedicels, nodes, and, rarely, inflorescences [88,153]. Broertjes and colleagues, in the late 1970s, had first published the in vitro regeneration of shoots from explants non-meristem plants as a criterion to generate non-chimeric mutants in chrysanthemums. According to Jo and Kim [154], the frequency of variation is influenced by several parameters. The most important types are the irradiation type and dose, linear energy transfer (LET), and the kind of tissue being irradiated.

Recently, ovaries have been used for breeding mutant *Chrysanthemum* by Miler and Muszczyk, Wang et al., and Miler and Muszczyk [14,155,156]. Ovaries were useful targets for radiation. The characteristics of the ovaries to be used as explants for breeding mutant *Chrysanthemum* include location within the inflorescence, gathering them on one side of the plane, and having a high ability of regeneration [157]. The in vitro regeneration of ovaries of two *Chrysanthemum* cultivars, ‘Profesor Jerzy’ and ‘Karolina,’ were done after radiation with high-energy photons (total doses of 5, 10, and 15 Gy) and high-energy electrons (total dose of 10 Gy) [14]. They demonstrated that irradiated ovaries from the whole inflorescences of *Chrysanthemum morifolium* (Ramat.) could be used efficiently in breeding programs for which the mother variety was regenerated in vitro effectively, having a strong impact on the regeneration efficiency of the genotype. The cultivar ‘Karolina’ formed only seven shoots, while the cultivar ‘Professor Jerzy’ produced 428 shoots. The regeneration rate decreased with the increased irradiation dose. Explants exposed to 10 Gy of high-energy electrons and 15 Gy of high-energy photons showed the lowest response. A total dose of 10 Gy of high-energy photons was administered (beam energy at 6 MeV and dose rate at 3.19 Gy per min), which was most effective in influencing the stable shape and color changes in inflorescences. They did not exhibit any adverse side effects like a delay or increased culture duration due to delayed blooming [14].

## 7. The Acclimatization Stage

Acclimatization under nursery conditions is highly critical for successful micropropagation techniques, wherein the plants are typically kept in high-humidity environments for a few days before transferring them to the greenhouse [60]. During the acclimatization stage, plants are subjected to various hazardous environmental influences, including microbial infections (mostly fungi and bacteria), temperature fluctuations, low humidity, and inadequate nutrition, all of which significantly reduce plant survival rates. However, there is in vitro control of plant growth [158]. In addition, when plant organs are transferred to ex vivo environments, physiological modulations within the organs result in morphological and anatomical defects. Plant stomata do not function correctly; roots are weak, and the epidermal layer is thin [159]. To develop methods that improve plant survival, growth, and development in greenhouses, it is essential to recognize the physiological and biochemical changes that occur in plants during acclimation [20]. To acclimate, the plants must develop leaf cuticles before being removed from tissue culture. The growing environment’s humidity should be reduced to enable the plants to develop a sturdy cuticle layer [4]. 

## 8. Alternative Light Sources in the Greenhouse

Light-emitting diodes (LEDs) can take the place of standard fluorescent lights to cut down on energy costs. In some studies, LEDs are more suitable fluorescent lights for in vitro and ex vitro study [160]. Two biological research areas of photosynthesis [161,162] and morphogenesis [161] utilize LEDs [162,163]. Light intensity, quality, spectrum, photoperiod, lighting direction, and photoperiod are the factors that affect its response [164]. Greenlight—G (565 nm), blue light—B (450 nm), red light—R (660 nm), and yellow light—Y (590 nm) [165] are all considered alternatives to traditional fluorescence lamps (FL) as light sources for micropropagation [166]. In greenhouses and tissue culture, the light sources (B) can control light intensity, CO_2_ percentage, relative humidity, temperature, chloroplast aggregation, and open stomata to promote plant growth and development [166,167]. In plants, the synthesis of chlorophyll was also aided by blue light [168]. The growth of greenhouse plant *C. grandiflorum* ‘Coral Charm’ was observed taking blue to red LEDs in various ratios. Plants were stunted in growth when exposed to 40% blue + 60% red light, while plants exposed to 100% red light had the lowest overall biomass. Stomatal conductance was higher in all red + blue LED ratios than in control, even though photosynthesis was unaffected. The levels of flavonoids were lowest when exposed to only red light, but the levels of flavonoids and phenolic acids were higher in treatments that used a high blue light proportion. The morphology of plants may benefit from these discoveries in the future [169]. In addition, *Chrysanthemum* shoots’ necrosis could be seen in the micropropagation (MR) system under blue light and yellow light, as mentioned by Tung et al. [20]. According to Lichtentaler and Wellburn [170], the wavelengths of the Y (590 nm) and G (565 nm) in maximum absorption spectrophotometers do not match those of chlorophyll a (662 nm) and chlorophyll b (645 nm). Blue—B (450 nm) LEDs had the maximum cytochrome and carotenoid absorption. *Chrysanthemum* shoots grown under red light were slender, yellowish, and had fresh weight (0.38 g). In contrast, those grown under B and R had chlorophyll a (15.87 gg1), chlorophyll b (8.97 gg1), and chlorophyll a + b (24.84 gg1) levels that were lower than those grown under B and R (10:90, 20:80, 30:70, 40:60, and 50:50). The combination of B and R at a 30:70 ratio improved plant growth and development and seedling quality. On the other side, uneven light-intensity distribution on the culture shelves is a problem with both conventional lighting systems and several commercially available LED lighting systems. LEDs are employed in some systems to increase plant quality; among these lighting systems is UNIPACK, which improves space, wavelength, and lighting efficiency. UNIPACK’s usage of several wires to give direct current to LED boards made the system complex [28]. In 2007, MIT (Massachusetts Institute of Technology) tested wireless power transfer without cables [171]. In that study, a new LED system was designed combining 30:70 blue–red LEDs and wireless power transmission. According to Nam et al. [28] and Nhut et al. [29], the considerable improvement in the survival rate of chrysanthemum seedlings achieved under LED lighting systems compared to those grown under fluorescent lighting systems was shown in both the length and dry weight of chrysanthemum seedlings being more significant in the LP and WPT-LP conditions compared to the FL condition. In addition, after 16 weeks in soil, chrysanthemum seedlings continued growing until flower bud formation when exposed to the combination of red and blue LED in three systems; fluorescent LED tube, LP, and WPT-LP.

## 9. Future Perspectives

Elite varieties can be propagated in vitro to produce sufficient planting materials that are true to type and would otherwise be difficult to come by. New discovered *Chrysanthemum* varieties could be introduced as soon as possible due to the rapid rate of in vitro propagation. Optimal media use, plant growth regulators used properly, and an appropriate in vitro culture methodology can help in the genetic engineering of *Chrysanthemum.* This will enhance trading in chrysanthemums, a profitable business, and a valuable scientific endeavor. 

## Figures and Tables

**Table 1:** Summary of recent studies focused on the optimal cultured medium in vitro for chrysanthemum proliferation.

Plant Sp.	Explant Type	Medium Content	Responding	Survival Response	References
*Chrysanthemum morifolium*	Segments of nodal nodules with a single axillary bud	Full strength MS basal medium + BAP (0.1 mg/L) + sucrose (30 g/L) + agar (7 g/L *w*/*v*).	Bud induction	96%	[46]
		-MS medium + BAP (0.5 mg/L)	Shoot multipication		
		-MS medium + 25 g/L psyllium husk + 20 g/L market sugar + RO	Shoot multiplication		
		1/2 strength MS medium + 0.6% agar + 20 g/L market sugar + 0.50 mg/L IBA	Root formation		
*Chrysanthemum × grandiflorum* Ramat. Kitam. cv. Capitola	Ligulate florets	-MS (Medium) + calcium and iron by half + 0.4 mg/L thiamine + 10 g/L sucrose, (6 weeks) 3 mg/L KIN + 0.5 mg/L (IAA) + 0.8% (*w*/*v*) agar Then (6 weeks) 1 mg/L KIN + 0.5 mg/L (IAA) + 0.8% (*w*/*v*) agar.	Shoot multiplication 51.26%	N/A	[94]
*Chrysanthemum morifolium* cv.ziyan	Stems with axillary bud	-MS + 6-BA 2.0 mg·L^−1^ + NAA 1.0 mg·L^−1^.	Callus induction 100% and bud differentiation 92.22%.	N/A Survival 100%	[39]
		-1/2 MS + NAA 0.2 mg·L^−1^	Rooting 100% and average root number of 15.50		
*C.morifolium* cv.niu9722	Stems with axillary bud	-MS + 6-BA 2.0 mg·L^−1^ + NAA 0.5 mg·L^−1^	Callus induction 100%+ bud differentiation 45.59%		
		MS + 6-BA 2.0 mg·L^−1^ + NAA 0.5 mg·L^−1^	Stem proliferation		
		1/2 MS + NAA 0.3 mg·L^−1^	Rooting 100% + average root number of 14.87		
*Chrysanthemum morifolium* Ramat cv., “Pasopati”	Leaf explants	MS + 1.0 mg L^−1^ + 0.5 mg L^−1^ BAP	Callus induction (1.55 g biomass weight)	N/A	[95]
		MS + NAA (0.5 mg L^−1^) + BAP (0.5 mg L^−1^)	Shoot initiation (30 days after planting) + N. of shoots (5) + shoot length (2.9 cm)		
		MS + 2iP (0.5 mg L^−1^) and BAP (2.0 mg L^−1^)	Shoot imitation after after 30 days + shoot length (1.88 cm) + shoot Num. (1)		
		MS + BAP 0.5 mg L^−1^	Num.shoot (3)		
*Chrysanthemum* cv. Shinma	Leaf explant	MS + 0.5 BA mg/L + 0.5 NAA mg/L + 3 g L^−1^ of Gelrite under the 16 h photoperiod (37 L mol m^−2^ s^−1^) for 45 days	Shoot regeneration 60%	95%	[34]
		MS + 0.2 mg L^−1^ IBA under the 16 h photoperiod (37 L mol m^−2^ s^−1^) for 45 days	No. of roots/explant (12) + root length (10.7)		
*Chrysanthemum morifolium* Ramat	Stem explant	SH basal medium + 1 mg/L IBA + 30 g/L sucrose + 3 g/L gelrite	The highest root no. (5.7)	100% survival	[19]
		SH basal medium + 1 mg/L IAA + 30 g/L sucrose + 3 g/L gelrite	The highest root length (36.2)		
*Chrysanthemum morifolium*	Nodal segments	MS medium + 1.0 mg/L BAP + 0.1 mg/L IAA	90% shoot initiation and 5.5 cm average length of shoot per explant	N/A	[96]
		MS medium + 1.0 mg/L BAP	93% shoot proliferation, 5.7 cm average lengths of shoot per explant and 4.4 nodes per explant		
		½ MS medium + 0.2 mg/L IBA	90% rooted micro cuttings; 9 cm length of root/explant + 11.8 no./explant		
*Chrysanthemum morifolium*	Nodal segment	MS + BA 2.0 mg/L	Shoot induction (80.00%) + shoot no. 3.00 at 28 DAI (days after induction)	80% in shade condition and 75% in open atmospheric condition.	[45]
		MS medium + BA 2.0 mg/L + 2,4-D 1.0 mg/L	Callus induction (76.00%) + shoot no. (3.20) at 40 DAI		
		MS medium + BA 2.0 mg/L + IAA 1.0 mg/L	Shoot length 3.66 cm at 28 DAI		
		1/2MS + IAA 0.5 mg/L	Root induction (80.00%) within 13 days		
		1/2MS medium + BA 3.0 mg/L + IAA 1.5 mg/L	Root induction (76%) within 12.20 days		
		1/2MS medium + BA 2.0 mg/L + IAA 1.0 mg/L	Root no 4.20 at 28 DAI		
		1/2MS medium + 1.5 mg/L IAA	Root No. (5.40) at 28 DAI		
*(Chrysanthemum × grandiflorum*/ Ramat./Kitam.) ‘Alchimist’	Leaf explants with or without callus	MS medium + 11.42 μM IAA + 2.66 μM BA + Irradiation conditions (MW = power of 800 W·cm^−2^ and the frequency of 2.45 GHz)	40% adventitious shoot	Acclimization 100%	[97]
		½MS medium + half-strength macronutrients + (11.42 μM) of IAA for 10 days	Rooting 100%		
*Chrysanthemum moliforium*	Shoots (3 cm in length)	MR: ½ MS, 30 g L^−1^ sucrose, pH 5.8 8 g L^−1^ agar + 7.5 ppm AgNP	MO: After 15 weeks in the greenhouse, flowering 100%	After 4 weeks well adapted and rapidly grown	[20]
		MO: ½ MS, without sugar, pH 5.8 ½, nylon fill			
*Chrysanthemum morifolium* CV. (Delistar White)	Ray florets	MS medium + BAP 1.0 mg/L + NAA 0.5 mg/L + sucrose 30 g/L + agar 5.5 g/L	-Callus induction -Shoot formation	N/A	[30]
		1/2 strength (MS medium) + NAA 0.1 mg/L + sucrose 15 g/L + agar 5.5 g/L	Root induction		
*Chrysanthemum (Dendranthema × grandiflorum)* “Hornbill Dark”	Leaf segments	MS medium + 2 mg·L^−1^ 2,4-D + 2 mg·L^−1^ BAP	100% callogenesis rate + 95.56% callogenesis rate + (9.73) somatic embryo number	N/A	[98]
*Chrysanthemum (Dendranthema × grandiflorum)* “Hornbill Dark”	Leaf explant	(MS) medium consisted of (3% sucrose, 0.7% agar, and 100 mg/L Myo-inositol.) + 9.09 μM 2,4-D + 4.65 μM BAP + 20 μM SNP	Callogenesis rate (100%), embryogenes rate (100%), and the number of somatic embryos per explant (57.8)	N/A	[99]
(*Chrysanthemum × morifolium*/Ramat.) CV. Profesor Jerzy/ ‘Karolina	Ovaries from Irradiated inflorescence	Induction medium for 12 weeks: 1.0 mg dm^−3^ (BAP) + 1.0 mg dm^−3^ (2,4-D)	33.3% (*‘Karolina*) induction callus	89.18% survival for Profesor Jerzy	[14]
		Regeneration medium for 18 weeks: 2.0 mg dm^−3^ kinetin + 1.0 mg dm^−3^ + (IAA) and 4.0 mg dm^−3^ glycine + pH 5.8	Shoot induction (66.6% shoots for (Profesor Jerzy)		
		Rooting medium: (MS based, supplemented with 2.0 mg dm^−3^ IAA, pH 5.8			
*Chrysanthemum indicum* L.	Single nodes from shoots	4 mg·L^−1^ Kin + 0.6 mg·L^−1^ IBA + (MS)	Shoot induction	N/A	[100]
		1/2 strength MS + 0.1 IBA	Root induction		
*Ch. moliforium*	Leaves (Four-week-old)	MS medium + AgNP(4 PPM), 30 g L^−1^ sucrose + 8 g L^−1^ agar. + 0.2 mg L^−1^ (BA)	Shoot regeneration 100% after 4 week of culture	100%	[62]

## Data Availability

Not applicable.

## References

[1] Azadi P., Bagheri H., Nalousi A.M., Nazari F., Chandler S. (2016). Current Status and Biotechnological Advances in Genetic Engineering of Ornamental Plants. Biotechnol. Adv..

[2] Getu M. (2009). Ethiopian Floriculture and Its Impact on the Environment. Mizan Law Rev..

[3] Arora J.S. (1990). Introductory Ornamental Horticulture.

[4] Spaargaren J., Geest G.V., Van Huylenbroeck J. (2018). Chrysanthemum. Ornamental Crops.

[5] Ryu J., Nam B., Kim B.R., Kim S.H., Jo Y.D., Ahn J.W., Han A.R., Kim J.B., Jin C.H., Han A.-R. (2019). Comparative Analysis of Phytochemical Composition of Gamma-Irradiated Mutant Cultivars of *Chrysanthemum Morifolium*. Molecules.

[6] Sassi A.B., Harzallah-Skhiri F., Bourgougnon N., Aouni M. (2008). Antimicrobial Activities of Four Tunisian Chrysanthemum Species. Indian J. Med. Res..

[7] Marongiu B., Piras A., Porcedda S., Tuveri E., Laconi S., Deidda D., Maxia A. (2009). Chemical and Biological Comparisons on Supercritical Extracts of *Tanacetum cinerariifolium* (Trevir) Sch. Bip. with Three Related Species of Chrysanthemums of Sardinia (Italy). Nat. Prod. Res..

[8] Collins R.A., Ng T.B., Fong W.P., Wan C.C., Yeung H.W. (1997). A Comparison of Human Immunodeficiency Virus Type 1 Inhibition by Partially Purified Aqueous Extracts of Chinese Medicinal Herbs. Life Sci..

[9] (2014). AIPH International Statistics Flowers International Statistics Flowers and Plants 2014.

[10] Kulus D. (2015). Selected Aspects of Ornamental Plants Micropropagation in Poland and Worldwide. Nauk. Przyr..

[11] Kumar R.K., Singh K.P., Raju D.V.S. (2015). Effect of Different Strains of Arbuscular Mycorrhizal Fungi (AMF) on Macro and Micro Nutrient Uptake in Micropropagated Chrysanthemum Plantlets. Vegetos.

[12] Rout G.R., Das P. (1997). Recent Trends in the Biotechnology of Chrysanthemum: A Critical Review. Sci. Hortic..

[13] Datta S.K. (2020). Induced Mutations: Technological Advancement for Development of New Ornamental Varieties. Nucleus.

[14] Miler N., Iwona J., Jakubowski S., Winiecki J. (2021). Ovaries of Chrysanthemum Irradiated with High-Energy Photons and High-Energy Electrons Can Regenerate Plants with Novel Traits. Agronomy.

[15] Catalano C., Carra A., Carimi F., Motisi A., Abbate L., Sarno M., Carrubba A. (2022). Long-Term Field Evaluation of Conventional vs. Micropropagated Plants of Chrysanthemum cinerariifolium. Agronomy.

[16] Hesami M., Naderi R., Tohidfar M., Miler N., Iwona J., Jakubowski S., Winiecki J., Hesami M., Naderi R., Tohidfar M. (2014). Effects of Growth Regulators and Genotypes on Pyrethrum in Vitro. Sci. Hortic..

[17] Lim K.B., Kwon S.J., Lee S.I., Hwang Y.J., Naing A.H. (2012). Influence of Genotype, Explant Source, and Gelling Agent on in Vitro Shoot Regeneration of Chrysanthemum. Hortic. Environ. Biotechnol..

[18] Naing A.H., Jeon S.M., Han J.S., Lim S.H., Lim K.B., Kim C.K. (2014). Factors Influencing in Vitro Shoot Regeneration from Leaf Segments of Chrysanthemum. Comptes Rendus-Biol..

[19] Chae S.C. (2016). Influence of Auxin Concentration on in Vitro Rooting of *Chrysanthemum Morifolium Ramat*. Biosci. Biotechnol. Res. Asia.

[20] Tung H.T., Nam N.B., Huy N.P., Luan V.Q., Hien V.T., Phuong T.T.B., Nhut D.T. (2018). A System for Large Scale Production of Chrysanthemum Using Microponics with the Supplement of Silver Nanoparticles under Light-Emitting Diodes. Sci. Hortic..

[21] Kim S.H., Kim Y.S., Jo Y.D., Kang S.Y., Ahn J.W., Kang B.C., Kim J.B. (2019). Sucrose and Methyl Jasmonate Modulate the Expression of Anthocyanin Biosynthesis Genes and Increase the Frequency of Flower-Color Mutants in Chrysanthemum. Sci. Hortic..

[22] Rahmy N., Thomy Z., Yunita, Harnelly E. (2019). The Effect of Some of Coconut Water Concentration in Artificial Media to Chrysanthemum Growth (*Dendranthema Grandiflora*) by in Vitro. J. Nat..

[23] Jahan M.T., Islam M.R., Islam S.S., Das P., Islam M.M., Kabir M.H., Mamun A.N.K. (2021). Clonal Propagation of *Chrysanthemum Morifolium Ramat* Using Various Explants Obtained from Field Grown Plants. GSC Biol. Pharm. Sci..

[24] Kulus D., Zalewska M. (2014). In Vitro Plant Recovery from Alginate-Encapsulated *Chrysanthemum × Grandiflorum* (Ramat.) Kitam. Shoot Tips. Propag. Ornam. Plants.

[25] Deein W., Thepsithar C., Thongpukdee A. (2013). In Vitro Culture Medium Sterilization by Chemicals and Essential Oils without Autoclaving and Growth of Chrysanthemum Nodes. World Acad. Sci..

[26] Zalewska M., Tymoszuk A., Miler N. (2011). New Chrysanthemum Cultivars as a Result of in Vitro Mutagenesis with the Application of Different Explant Types. Acta Sci. Pol. Hortorum Cultus.

[27] Dwimahyani I., Widiarsih S. (2010). The Effects of Gamma Irradiation on the Growth and Propagation of In-Vitro Chrysanthemum Shoot Explants (Cv. Yellow Puma). At. Indones..

[28] Nam N.B., Huy N.P., Luan V.Q., Tung H.T., Nhut D.T. (2016). Application of Wireless Power Transmission Led Lighting System in Propagation of Chrysanthemum and Strawberry. Planta Daninha.

[29] Nhut D.T., Nam N.B., Tung H.T. (2022). Wireless Light-Emitting Diode System for Micropropagating Chrysanthemum and Strawberry. Plant Tissue Culture: New Techniques and Application in Horticultural Species of Tropical Region.

[30] Datta S.K. (2019). Need Based Tissue Culture in Floriculture: Asuccess Story. J. Plant Sci. Res..

[31] Kyte L., Kleyn J., Scoggins H., Bridgen M. (2013). Plants from Test Tubes: An Introduction to Micropropogation.

[32] Anderson W.C. (1980). Mass Propogation by Tissue-Culture-Principles and Techniques. Sci. Educ. Adm. Publ..

[33] Thrope T.A., Rodríguez R., Tamés R.S., Durzan D.J. (1990). Organogenesis in Vitro: Structural, Physiological, and Biochemical Aspects. Plant Aging. NATO ASI Series.

[34] Naing A.H., Il Park K., Chung M.Y., Lim K.B., Kim C.K. (2016). Optimization of Factors Affecting Efficient Shoot Regeneration in *Chrysanthemum* Cv. Shinma. Rev. Bras. Bot..

[35] Jevremovic S., Subotic A., Miljkovic D., Trifunovic M., Petric M., Cingel A. (2011). Clonal Fidelity of Chrysanthemum Cultivars after Long Term Micropropagation by Stem Segment Culture. Acta Hortic..

[36] Waseem K., Jilani M.S., Khan M.S. (2009). Rapid Plant Regeneration of Chrysanthemum (*Chrysanthemum Morifolium* L.) through Shoot Tip Culture. Afr. J. Biotechnol..

[37] Wankhede K.N., Narkhede M.N., Shivankar R.S., Rathod T.H. (2000). Callus Induction and Micropropagation Studies in Chrysanthemum. Ann. Plant Physiol..

[38] Himstedt J.P., Jacobsen H.J., Fischer K. (2000). Shoot regeneration from stem and leaf explants of Chrysanthemum (*Dendranthema × grandiflorum*). Acta Hortic..

[39] Liu C.X., Ma X., Dong F.L., Zhou Y.W. (2015). Establishment of Regeneration System of *Chrysanthemum Morifolium* ‘ziyan’ and ‘niu 9722’. Pratacultural Sci..

[40] Petty L.M., Harberd N.P., Carré I.A., Thomas B., Jackson S.D. (2003). Expression of the Arabidopsis Gai Gene under Its Own Promoter Causes a Reduction in Plant Height in Chrysanthemum by Attenuation of the Gibberellin Response. Plant Sci..

[41] Sauvadet M.-A., Brochard P., Boccon-Gibod J. (1990). A Protoplast-to-Plant System in Chrysanthemum: Differential Responses among Several Commercial Clones. Plant Cell Rep..

[42] Fu-Yun L. (2010). Tissue Culture and Rapid Propagation of *Chrysanthemum Morifolium*. Plant Phisiol. Commun..

[43] Jevremovic S., Radojevic L.J. (2004). Mass Production of Different Chrysanthemum (*Chrysanthemum Morifolium*) Cultivars by Culture in Vitro. J. Sci. Agric. Res..

[44] Wang X., Zeng L., Peng Y., Xi C., Lin D. (2013). Studies on Rapid-Micropropagation Technology of Different Chrysanthemum Cultivars. J. Shanghai Jiaotong Univ. Sci..

[45] Khan S.I. (2017). In Vitro Planet Regeneration of Chrtsanthemum (*Chrysanthemum Morifolium*). Ph.D. Thesis.

[46] Pant M., Lal A., Jain R. (2015). A Simple Cost Effective Method for Mass Propagation of *Chrysanthemum Morifoliumi* and Antibacterial Activity Assessment of in Vitro Raised Plantlets. J. Appl. Pharm. Sci..

[47] Waseem K., Jilani M., Jaskani M., Khan M., Kiran M., Khan G. (2011). Significance of Different Plant Growth Regulators on the Regeneration of Chrysanthemum Plantlets (*Dendranthema Morifolium* L.) through Shoot Tip Culture. Pak. J. Bot..

[48] Lacostales L.E., Acedo V.Z. (2015). Single Nodal Cutting Propagation of Tissue Culture-Derived Chrysanthemum (*Chrysanthemum Morifolium* Ramat.). Philipp. J. Crop Sci. (Philipp.).

[49] Zalewska M., Miler N., Wenda-Piesik A. (2010). Effect of in Vitro Topophysis on the Growth, Development, and Rooting of Chrysanthemum Explants (*Chrysanthemum Grandiflorum*/Ramat./Kitam). J. Hortic. Sci. Biotechnol..

[50] Barakat M.N., AbdelFattah R.S., Badr M., El-Torky M.G. (2010). In Vitro Culture and Plant Regeneration Derived from Ray Florets of *Chrysanthemum Morifolium*. Afr. J. Biotechnol..

[51] TeixeiradaSilva J.A., Kulus D. (2014). Chrysanthemum Biotechnology: Discoveries from the Recent Literature. Folia Hortic..

[52] Datta S.K. (2015). Indian Floriculture: Role of CSIR.

[53] Miler N., Zalewska M. (2014). Somaclonal Variation of Chrysanthemum Propagated in Vitro from Different Explants Types. Acta Sci. Pol. Hortorum Cultus.

[54] Kengkarj P., Smitamana P., Fujime Y. (2008). Assessment of Somaclonal Variation in Chrysan-Themum (*Dendranthema Grandiflora Kitam.*) Using RAPD and Morphological Analysis. Plant Tissue Cult. Biotechnol..

[55] Murashige T., Skoog F. (1962). A Revised Medium for Rapid Growth and Bio Assays with Tobacco Tissue Cultures. Physiol. Plant..

[56] Lee T., Huang M.E.E., Pua E.-C.C. (1997). High Frequency Shoot Regeneration from Leaf Disc Explants of Garland Chrysanthemum (*Chrysanthemum Coronarium* L.) in vitro. Plant Sci..

[57] Tyagi R.K., Agrawal A., Mahalakshmi C., Hussain Z., Tyagi H. (2007). Low-Cost Media for in Vitro Conservation of Turmeric (*Curcuma Longa* L.) and Genetic Stability Assessment Using RAPD Markers. Vitr. Cell. Dev. Biol..

[58] Atici T., Khawar K.M., Ozel C.A., Katircioglu H., Ates M.A. (2008). Use of Psyllium (Isubgol) Husk as an Alternative Gelling Agent for the Culture of Prokaryotic Microalgae (Cyanobacteria) Chroococcus Limneticus Lemmermann and Eukaryotic Green Microalgae (Chlorophyta) Scenedesmus Quadricauda (Turpin) Brebisson. Afr. J. Biotechnol..

[59] Rao S.N.P., Kumar A.Y.R. (2011). Effects of Antioxidants and Gelling Agents on Regeneration, in Vitro Conservation and Genetic Stability of *Bacopa Monnieri* (L.) Pennell. Int. J. Ayurvedic Herb. Med..

[60] George E.F., Hall M.A., Klerk G.-J., George E.F., Hall M.A., Klerk G.D. (2008). De The Components of Plant Tissue Culture Media II: Organic Additions, Osmotic and PH Effects, and Support Systems. Plant Propagation by Tissue Culture.

[61] Sahu J., Sahu R.K. (2013). A Review on Low Cost Methods for in Vitro Micropropagation of Plant through Tissue Culture Technique. Pharm. Biosci. J..

[62] Tung H.T., Bao H.G., Cuong D.M., Ngan H.T.M., Hien V.T., Luan V.Q., Nhut D.T. (2021). Silver Nanoparticles as the Sterilant in Large-Scale Micropropagation of Chrysanthemum. Vitr. Cell. Dev. Biol.-Plant.

[63] Chen C. (2016). Cost Analysis of Plant Micropropagation of Phalaenopsis. Plant Cell Tissue Organ Cult..

[64] Wang X.-J., Hsiao K.-C. (1995). Sugar Degradation during Autoclaving: Effects of Duration and Solution Volume on Breakdown of Glucose. Physiol. Plant..

[65] Brondani G.E., de Oliveira L.S., Bergonci T., Brondani A.E., França F.A.M., da Silva A.L.L., Goncalves A.N. (2013). Chemical Sterilization of Culture Medium: A Low Cost Alternative to in Vitro Establishment of Plants. Sci. For..

[66] Pais A.K., da Silva A.P., de Souza J.C., Teixeira S.L., Ribeiro J.M., Peixoto A.R., da Paz C.D. (2016). Sodium Hypochlorite Sterilization of Culture Medium in Micropropagation of Gerbera Hybrida Cv. Essandre. Afr. J. Biotechnol..

[67] Vargas D.P., Formoso R.S., Dutra L.F., Mayer N.A., Santos J.D., Ueno B. (2016). Chemical Sterilization of in vitro Culture for Peach Rootstock. Proceedings of the Colloquium Agrariae.

[68] Cardoso J.C., Gerald L.T.S., da Silva J.A.T. (2018). Micropropagation in the Twenty-First Century. Micropropagation in the Twenty-First Century, in Plant Cell Culture Protocols.

[69] Tian C., Xie Z., Zhao Y., Zhang Z., Xue T., Sheng W., Zhao F., Duan Y. (2022). Microgram-Grade Concentration of Chlorine Dioxide Induces One-Step Plant Regeneration in Chrysanthemum. Vitr. Cell. Dev. Biol.-Plant.

[70] Tymoszuk A. (2014). Application of Silver and Copper Nanocolloids in Disinfection of Explants in Chrysanthemum In Vitro Cultures. Book of Abstracts, Proceedings of the NanoPL.

[71] Sarmast M., Salehi H., Khosh-Khui M. (2011). Nano Silver Treatment Is Effective in Reducing Bacterial Contaminations of *Araucaria Excelsa* R. Br. Var. Glauca Explants. Acta Biol. Hung..

[72] Sarmast M.K., Salehi H. (2016). Silver Nanoparticles: An Influential Element in Plant Nanobiotechnology. Mol. Biotechnol..

[73] Zhou Y., Wang Y., Song Y., Gao Z., Liu Y., Fan L., Hu Q., Gao S. (2014). Stem Apex Detoxification Culture Markedly Improved Several Physiological Characters of Chrysanthemum ‘YUTAI. ’ Plant Cell. Tissue Organ Cult..

[74] Hahn E.J., Lee Y.B., Ahn C.H., Kozai T. A New Method on Mass-Production of Micropropagated Chrysanthemum Plants Using Microponic System in Plant Factory. Proceedings of the International Symposium on Plant Production in Closed Ecosystems.

[75] Hahn E.J., Bae J.H., Lee Y.B. (1998). Growth and Leaf-Surface Characteristics of Chrysanthemum Plantlets between Micropropagation and Microponic System. J. Korean Soc. Hortic. Sci. (Korea Repub.).

[76] Hahn E.-J., Bae J.-H., Lee Y.-B. (2000). Growth and Photosynthetic Characteristics of Chrysanthemum Plantlets as Affected by PH and EC of the Nutrient Solution in Microponic Culture. Hortic. Environ. Biotechnol..

[77] Tung H.T., Ngan H.T.M., Phuong T.T.B., Nhut D.T. (2022). Microponic Culture System in the Propagation of Some Plants. Plant Tissue Culture: New Techniques and Application in Horticultural Species of Tropical Region.

[78] Thepsithar C., Thongpukdee A., Daorat A. (2013). Sterilisation of in Vitro Culture Medium of Chrysanthemum by Plant Essential Oils without Autoclaving. Int. J. Bioeng. Life Sci..

[79] Russell A.D. (2003). Similarities and Differences in the Responses of Microorganisms to Biocides. J. Antimicrob. Chemother..

[80] Shukla P.K., Misra P., Kole C. (2016). Plant Nanotechnology: Principles and Practices.

[81] Sahu N., Soni D., Chandrashekhar B., Sarangi B.K., Satpute D., Pandey R.A. (2012). Synthesis and Characterization of Silver Nanoparticles Using *Cynodon Dactylon* Leaves and Assessment of Their Antibacterial Activity. Bioprocess Biosyst. Eng..

[82] Savithramma N., Ankanna S., Bhumi G. (2012). Effect of Nanoparticles on Seed Germination and Seedling Growth of *Boswellia Ovalifoliolata* an Endemic and Endangered Medicinal Tree Taxon. Nano Vis..

[83] Tymoszuk A., Miler N. (2019). Silver and Gold Nanoparticles Impact on in Vitro Adventitious Organogenesis in Chrysanthemum, Gerbera and Cape Primrose. Sci. Hortic..

[84] Dimkpa C.O., McLean J.E., Britt D.W., Anderson A.J. (2012). Bioactivity and Biomodification of Ag, ZnO, and CuO Nanoparticles with Relevance to Plant Performance in Agriculture. Ind. Biotechnol..

[85] Tung H.T., Bao H.G., Buu N.Q., Chau N.H., Nhut D.T. (2022). The Use of Silver Nanoparticles as a Disinfectant and Media Additive in Plant Micropropagation. Plant Tissue Culture: New Techniques and Application in Horticultural Species of Tropical Region.

[86] Azcón-Aguilar C., Barea J.M. (1997). Arbuscular Mycorrhizas and Biological Control of Soil-Borne Plant Pathogens—An Overview of the Mechanisms Involved. Mycorrhiza.

[87] Zandavalli R.B., Dillenburg L.R., de Souza P.V.D. (2004). Growth Responses of *Araucaria Angustifolia* (Araucariaceae) to Inoculation with the Mycorrhizal Fungus Glomus Clarum. Appl. Soil Ecol..

[88] Sohn B.K., Kim K.Y., Chung S.J., Kim W.S., Park S.M., Kang J.G., Rim Y.S., Cho J.S., Kim T.H., Lee J.H. (2003). Effect of the Different Timing of AMF Inoculation on Plant Growth and Flower Quality of Chrysanthemum. Sci. Hortic..

[89] Abdalla M.E., Abdel-Fattah G.M. (2000). Influence of the Endomycorrhizal Fungus *Glomus Mosseae* on the Development of Peanut Pod Rot Disease in Egypt. Mycorrhiza.

[90] Ruiz-Lozano J.M., Azcón R. (1995). Hyphal Contribution to Water Uptake in Mycorrhizal Plants as Affected by the Fungal Species and Water Status. Physiol. Plant..

[91] Johansson J.F., Paul L.R., Finlay R.D. (2004). Microbial Interactions in the Mycorrhizosphere and Their Significance for Sustainable Agriculture. FEMS Microbiol. Ecol..

[92] Parsons B.J. (2013). Sterilisation Procedures for Tissue Allografts. Stand. Cell Tissue Eng..

[93] Nahid J.S., Shyamali S., Kazumi H. (2007). High Frequency Shoot Regeneration from Petal Explants of *Chrysanthemum Morifolium Ramat.* in Vitro. Pak. J. Biol. Sci..

[94] Jerzy M., Zalewska M., Tymoszuk A. (2015). Effect of Kinetin on the Elongation of Adventitious Shoots Regenerated in vitro from Ligulate Florets in *Chrysanth*. × Grandiflorum Ramat. Kitam. Acta Hortic..

[95] Sjahril R., Haring F., Riadi M., Rahim M.D., Khan R.S., Amir A., Trisnawaty A.R. (2015). Performance of NAA, 2iP, BAP and TDZ on Callus Multiplication, Shoots Initiation and Growth for Efficient Plant Regeneration System in Chrysanthemum (*Chrysanthemum Morifolium* Ramat). Int. J. Agric. Syst. Perform..

[96] Labade G.B., Dale N.S., Umbarkar R.B., Gadhe S.K., Rote Y.N. (2016). In Vitro Regeneration of Chrysanthemum (*Chrysanthemum Morifolium* L.). Int. J. Inf. Res. Rev..

[97] Miler N., Kulus D. (2018). Microwave Treatment Can Induce Chrysanthemum Phenotypic and Genetic Changes. Sci. Hortic..

[98] Hesami M., Naderi R., Tohidfar M. (2020). Introducing a Hybrid Artificial Intelligence Method for High-Throughput Modeling and Optimizing Plant Tissue Culture Processes: The Establishment of a New Embryogenesis Medium for Chrysanthemum, as a Case Study. Appl. Microbiol. Biotechnol..

[99] Hesami M., Naderi R., Tohidfar M., Yoosefzadeh-Najafabadi M., Hesami M., Naderi R., Tohidfar M., Yoosefzadeh-Najafabadi M. (2020). Development of Support Vector Machine-Based Model and Comparative Analysis with Artificial Neural Network for Modeling the Plant Tissue Culture Procedures: Effect of Plant Growth Regulators on Somatic Embryogenesis of Chrysanthemum, as a Case Study. Plant Methods.

[100] Alsoufi A.S.M.M., Ahmed Z.S., Salim A.M. (2021). The Efficiency of Interaction between Cytokines and Auxins in Micropropagation of Chrysanthemum Plant (*Chrysanthemum Indicum L.*). IOP Conf. Ser. Earth Environ. Sci..

[101] Imtiaz M., Khattak A.M., Ara N., Iqbal A., Rahman H.U. (2014). Micropropagation of *Jartorpha Curcas L.* through Shoot Tip Explants Using Different Concentrations of Phytohormones. J. Anim. Plant Sci..

[102] Park S.H., Kim G.H., Jeong B.R. (2007). Adventitious Shoot Regeneration from Cultured Petal Explants of Chrysanthemum. Hortic. Environ. Biotechnol..

[103] Janarthanam B., Seshadri S. (2008). Plantlet Regeneration from Leaf Derived Callus of *Vanilla Planifolia* Andr. Vitr. Cell. Dev. Biol..

[104] Trigiano R.N., Gray D.J. (2004). Plant Development and Biotechnology.

[105] Gao Y., Zhao B., Ding G., Zhang Q. (2001). Shoot Regeneration from Stem and Leaf Explants of *Dendrathema Grandiflorum*. J. Beijing For. Univ..

[106] Kashif W., Khan M.Q., Jaffar J., Khan M.S. (2007). Impact of Different Auxins on the Regeneration of Chrysanthemum (*Dendranthema Morifolium*) through in Vitro Shoot Tip Culture. Pak. J. Agric. Res..

[107] Hoque M.I., Fatema M. (1995). In Vitro Multiple Shoot Regeneration in *Chrysanthemum Morifolium* Ramat. Plant Tissue Cult.

[108] Hossain S.N., Hakim L., Islam M.R., Munshi M.K., Hossain M. (2002). In Vitro Plant Regeneration of Apple (*Malus Domestica Borkh*). Bangladesh J. Bot..

[109] Jahan M.T., Islam M.R., Khan R., Mamun A.N.K., Ahmed G., Hakim L. (2009). In Vitro Clonal Propagation of Anthurium (*Anthurium Andraeanum L.*) Using Callus Culture. Plant Tissue Cult. Biotechnol..

[110] Lindiro C., Kahia J., Asiimwe T., Mushimiyimana I., Waweru B., Kouassi M., Koffi E., Kone S., Sallah P.Y. (2013). In Vitro Regeneration of Pyrethrum (*Chrysanthemum Cinerariaefolium*) Plantlets from Nodal Explants of in Vitro Raised Plantlets. Int. J. Appl. Or Innov. Eng. Manag..

[111] Haberer G., Kieber J.J. (2002). Cytokinins. New Insights into a Classic Phytohormone. Plant Physiol..

[112] Al-Jobouri A.H. (2020). Studying Some The Functional Properties of Tamarind *Tamarindus Indica* L. Mucilage. Al-Qadisiyah J. Agric. Sci..

[113] XiaoHan Y., Bo J., Yan Z., Ding M., XueMei T., Dewei Z. (1993). Inhancement of Direct Shoot Regeneration from Internode Segments of Chrysanthemum by Silver Nitrate. Proceedings of the International Symposium on Cultivar Improvement of Horticultural Crops; Part 3: Flowers 404.

[114] Cho U.-H., Kasha K.J. (1989). Ethylene Production and Embyogenesis from Anther Cultures of Barley (*Hordeum Vulgare*). Plant Cell Rep..

[115] Pierik R.L.M. (1997). In Vitro Culture of Higher Plants.

[116] Francis D., Sorrell D.A. (2001). The Interface between the Cell Cycle and Plant Growth Regulators: A Mini Review. Plant Growth Regul..

[117] Thomas T.D., Maseena E.A. (2006). Callus Induction and Plant Regeneration in *Cardiospermum Halicacabum* Linn. an Important Medicinal Plant. Sci. Hortic..

[118] Obukosia S.D., Kimani E., Waithaka K., Mutitu E., Kimani P.M. (2005). Effects of Growth Regulators and Genotypes on Pyrethrum in Vitro. Vitr. Cell. Dev. Biol..

[119] Azwin, Slregar I.Z., Supriyanto D. (2006). The Use of BAP and TDZ for Propagation of Agarwood (*Aquilaria Malaccensis* Lamk.). Media. Media Konserv..

[120] Murgayanti S.E., Wieny R.H., Rustiani S. (2010). Callus Formation and Plant Regeneration of Chrysanthemum Leaf Disks Explants through in Vitro. International. Int. Semin. Hortic. Support Food Secur..

[121] Smith R.H. (2012). Plant Tissue Culture: Techniques and Experiments.

[122] Rivai R.R., Helmanto H. (2015). Callus Induction of *Chrysanthemum Indicum* for Increasing Genetic Diversity from Somatic Cell. Pros. Semin. Nas. Masy. Biodiversitas Indones..

[123] Nasri F., Zakizadeh H., Vafaee Y., Mozafari A. (2018). In Vitro Propagation of Chrysanthemum: An Overview on Its Utility in Mutagenesis and Genetic Transformation Techniques. Agric. Res. Technol. Open Access J..

[124] Greenway M.B., Phillips I.C., Lloyd M.N., Hubstenberger J.F., Phillips G.C. (2012). A Nutrient Medium for Diverse Applications and Tissue Growth of Plant Species in Vitro. Vitr. Cell. Dev. Biol..

[125] Shinoyama H., Nomura Y., Tsuchiya T., Kazuma T. (2004). A Simple and Efficient Method for Somatic Embryogenesis and Plant Regeneration from Leaves of Chrysanthemum [*Dendranthema × Grandiflorum* (Ramat.) Kitamura]. Plant Biotechnol..

[126] Kereša S., Mihovilović A., Barić M., Židovec V., Skelin M. (2012). The Micropropagation of Chrysanthemums via Axillary Shoot Proliferation and Highly Efficient Plant Regeneration by Somatic Embryogenesis. Afr. J. Biotechnol..

[127] Mani T., Senthil K. (2011). Multiplication of Chrysanthemum through Somatic Embryogenesis. Asian J. Pharma Tech..

[128] Naing A.H., Kim C.K., Yun B.J., Jin J.Y., Lim K.B. (2013). Primary and Secondary Somatic Embryogenesis in *Chrysanthemum* Cv. Euro. Plant Cell Tissue Organ Cult..

[129] Tymoszuk A., Zalewska M., Lema-Rumińska J. (2014). Regeneration of Somatic Embryos from in Vitro Isolated Ligulate Florets of Chrysanthemum. Acta Sci. Pol. Hortorum Cultus.

[130] Cline M.G. (1991). Apical Dominance. Bot. Rev..

[131] Roest S., Bokelmann G.S. (1973). Vegetative Propagation of *Chrysanthemum Cinerariaefolium* in Vitro. Sci. Hortic..

[132] Kazeroonian N., Power J.B., Davey M.R. (2014). An Assessment of Somaclonal Variation on *Chrysanth.* Cv. “Rosomee Splendid”. Sci. Hortic..

[133] Schenk R.U., Hildebrandt A.C. (1972). Medium and Techniques for Induction and Growth of Monocotyledonous and Dicotyledonous Plant Cell Cultures. Can. J. Bot..

[134] Imtiaz M., Khattak A.M., Khan M.A., Jalal F., Hussain S., Said F., Bo H. (2019). Rapid in-Vitro Propagation of *Chrysanthemum Morifolium* through Shoot Bud Explants. Pak. J. Bot..

[135] Verma O.P. (2012). Standardization of Auxin Concentration for Root Induction in *Chrysanthemum Morifolium*. Adv. Appl. Sci. Res.

[136] Gunawan B., Braun S., Cortés M.J., Bergmann F., Karl C., Füzesi L. (1998). Characterization of a Newly Established Endometrial Stromal Sarcoma Cell Line. Int. J. Cancer.

[137] Basri Z. (2008). Multiplikasi Empat Varietas Krisan Melalui Teknik Kultur Jaringan. J. Agroland..

[138] Malik S., Chadhury R., Kalia R. (2005). Rapid in Vitro Multiplication and Conservation of *Garcinia Indica*: A Tropical Medicinal Tree Species. Sci. Hortic..

[139] Ilahi I., Jabeen M., Nazneen Sadaf S. (2007). Rapid Clonal Propagation of Chrysanthemum through Embryogenic Callus Formation. Pak. J. Bot..

[140] Tymoszuk A., Zalewska M. (2014). In Vitro Adventitious Shoots Regeneration from Ligulate Florets in the Aspect of Application in Chrysanthemum Breeding. Acta Sci. Pol. Hortorum Cultus.

[141] Lakshmi M.K., Patil S.R., Chakrapani K., Kalamkar V.B., Lende S.R. (2006). Studies on Callus Induction and Differentiation in Chrysanthemum (*Dendranthema Grandiflora*). J. Soils Crop..

[142] Matsumura A., Nomizu T., Furutani N., Hayashi K., Minamiyama Y., Hase Y. (2010). Ray Florets Color and Shape Mutants Induced by 12C5+ Ion Beam Irradiation in Chrysanthemum. Sci. Hortic..

[143] Nalini R., Anjana M.J., Arathi C.S., Aswathy M., Ayana B., Bhuvaneswari R. (2016). Effect of Growth Regulators on Micropropagation of Chrysanthemum (*Dendranthema Grandiflora* Ramat.) Scru. IRJ Agric.

[144] Zafarullah A., Ilyas S., Naz S., Aslam F., Manzoor F. (2013). Effect of Culture Media and Growth Regulators on In Vitro Propogation of *Chrysanthemum Indicum* L.. Pak. J. Sci..

[145] Hitmi A., Barthomeuf C., Sallanon H. (1999). Rapid Mass Propagation of *Chrysanthemum Cinerariaefolium* Vis. by Callus Culture and Ability to Synthesise Pyrethrins. Plant Cell Rep..

[146] Shelake R.M., Pramanik D., Kim J.-Y. (2019). Evolution of Plant Mutagenesis Tools: A Shifting Paradigm from Random to Targeted Genome Editing. Plant Biotechnol. Rep..

[147] CPVO Varieties Database. http://Cpvo.Europa.Eu.

[148] (2008). Protocol for Distinctness, Uniformity and Stability Test for Chrysanthemum × Morifolium (Ramat.), Community Plant Variety Office (CPVO). https://cpvo.europa.eu/sites/default/files/documents/chrysanthemum_2.pdf.

[149] Zhao M.-X., Sun H.-Y., Ji R.-R., Hu X.-H., Sui J.-M., Qiao L.-X., Chen J., Wang J.-S. (2013). In Vitro Mutagenesis and Directed Screening for Salt-Tolerant Mutants in Peanut. Euphytica.

[150] Cretescu I., Rodica C., Velicevici G., Ropciuc S., Buzamat G. (2013). Response of Barley Seedlings to Microwaves at 2.45 GHz. Anim. Sci. Biotechnol..

[151] Halmagyi A., Surducan E., Surducan V. (2017). The Effect of Low- and High-Power Microwave Irradiation on in Vitro Grown Sequoia Plants and Their Recovery after Cryostorage. J. Biol. Phys. Vol..

[152] Misra P., Datta S.K., Chakrabarty D. (2003). Mutation in Flower Colour and Shape of *Chrysanthemum Morifolium* Induced by-γ-Radiation. Biol. Plant..

[153] Yamaguchi H., Shimizu A., Hase Y., Tanaka A., Shikazono N., Degi K., Morishita T. (2010). Effects of Ion Beam Irradiation on Mutation Induction and Nuclear DNA Content in Chrysanthemum. Breed. Sci..

[154] Jo Y.D., Kim J.-B. (2019). Frequency and Spectrum of Radiation-Induced Mutations Revealed by Whole-Genome Sequencing Analyses of Plants. Quantum Beam Sci..

[155] Miler N., Muszczyk P. (2015). Regeneration of Callus and Shoots from the Ovules and Ovaries of Chrysanthemum in Vitro. Acta Hortic..

[156] Wang H., Dong B., Jiang J., Fang W., Guan Z., Liao Y., Chen S., Chen F. (2014). Characterization of in Vitro Haploid and Doubled Haploid *Chrysanthemum Morifolium* Plants via Unfertilized Ovule Culture for Phenotypical Traits and DNA Methylation Pattern. Front. Plant Sci..

[157] Teixeira da Silva J.A., Dobránszki J. (2014). Dissecting the Concept of the Thin Cell Layer: Theoretical Basis and Practical Application of the Plant Growth Correction Factor to Apple, Cymbidium and Chrysanthemum. J. Plant Growth Regul..

[158] Valero-Aracama C., Kane M.E., Wilson S.B., Vu J.C., Anderson J., Philman N.L. (2006). Photosynthetic and Carbohydrate Status of Easy-and Difficult-to-Acclimatize Sea Oats (*Uniola Paniculata* L.) Genotypes during in Vitro Culture and Ex Vitro Acclimatization. Vitr. Cell. Dev. Biol..

[159] Mathur A., Mathur A.K., Verma P., Yadav S., Gupta M.L., Darokar M.P. (2008). Biological Hardening and Genetic Fidelity Testing of Micro-Cloned Progeny of *Chlorophytum Borivilianum* Sant. et Fernand. Afr. J. Biotechnol..

[160] Gupta S.D., Jatothu B. (2013). Fundamentals and Applications of Light-Emitting Diodes (LEDs) in in Vitro Plant Growth and Morphogenesis. Plant Biotechnol. Rep..

[161] Tripathy B.C., Brown C.S. (1995). Root-Shoot Interaction in the Greening of Wheat Seedlings Grown under Red Light. Plant Physiol..

[162] Tennessen D.J., Singsaas E.L., Sharkey T.D. (1994). Light-Emitting Diodes as a Light Source for Photosynthesis Research. Photosynth. Res..

[163] Hoenecke M.E., Bula R.J., Tibbitts T.W. (1992). Importance of Blue’Photon Levels for Lettuce Seedlings Grown under Red-Light-Emitting Diodes. HortScience.

[164] Taiz L., Zeiger E. (2007). Plant Physiology.

[165] Steigerwald D.A., Bhat J.C., Collins D., Fletcher R.M., Holcomb M.O., Ludowise M.J., Martin P.S., Rudaz S.L. (2002). Illumination with Solid State Lighting Technology. IEEE J. Sel. Top. Quantum Electron..

[166] Bula R.J., Morrow R.C., Tibbitts T.W., Barta D.J., Ignatius R.W., Martin T.S. (1991). Light-Emitting Diodes as a Radiation Source for Plants. HortScience.

[167] Kinoshita T., Doi M., Suetsugu N., Kagawa T., Wada M., Shimazaki K. (2001). Phot1 and Phot2 Mediate Blue Light Regulation of Stomatal Opening. Nature.

[168] Akoyunoglou G., Anni H. (1984). Blue Light Effect on Chloroplast Development in Higher Plants. Blue Light Effects in Biological Systems.

[169] Ouzounis T., Fretté X., Rosenqvist E., Ottosen C.-O. (2014). Spectral Effects of Supplementary Lighting on the Secondary Metabolites in Roses, Chrysanthemums, and Campanulas. J. Plant Physiol..

[170] Lichtentaler H.K., Wellburn A.R. (1983). Determination of Total Carotenoids, Chlorophyll a and b of Leaf in Different Solvents. Biochem. Soc. Trans..

[171] Kurs A., Karalis A., Moffatt R., Joannopoulos J.D., Fisher P., Soljacic M. (2007). Wireless Power Transfer via Strongly Coupled Magnetic Resonances. Science. Science.

